# A Phylogenetic Perspective on the Evolution of Mediterranean Teleost Fishes

**DOI:** 10.1371/journal.pone.0036443

**Published:** 2012-05-08

**Authors:** Christine N. Meynard, David Mouillot, Nicolas Mouquet, Emmanuel J. P. Douzery

**Affiliations:** 1 Institut des Sciences de l′Evolution, UMR 5554-CNRS-IRD, Université de Montpellier II, Place Eugene Bataillon, CC065, Montpellier, France; 2 INRA, UMR CBGP (INRA/IRD/Cirad/Montpellier SupAgro), Campus international de Baillarguet, CS 30016, Montferrier-sur-Lez, France; 3 Ecosystèmes Lagunaires, UMR 5119 CNRS-UM2-IFREMER-IRD, Place Eugene Bataillon, Montpellier, France; University of Otago, New Zealand

## Abstract

The Mediterranean Sea is a highly diverse, highly studied, and highly impacted biogeographic region, yet no phylogenetic reconstruction of fish diversity in this area has been published to date. Here, we infer the timing and geographic origins of Mediterranean teleost species diversity using nucleotide sequences collected from GenBank. We assembled a DNA supermatrix composed of four mitochondrial genes (12S ribosomal DNA, 16S ribosomal DNA, cytochrome c oxidase subunit I and cytochrome b) and two nuclear genes (rhodopsin and recombination activating gene I), including 62% of Mediterranean teleost species plus 9 outgroups. Maximum likelihood and Bayesian phylogenetic and dating analyses were calibrated using 20 fossil constraints. An additional 124 species were grafted onto the chronogram according to their taxonomic affinity, checking for the effects of taxonomic coverage in subsequent diversification analyses. We then interpreted the time-line of teleost diversification in light of Mediterranean historical biogeography, distinguishing non-endemic natives, endemics and exotic species. Results show that the major Mediterranean orders are of Cretaceous origin, specifically ∼100–80 Mya, and most Perciformes families originated 80–50 Mya. Two important clade origin events were detected. The first at 100–80 Mya, affected native and exotic species, and reflects a global diversification period at a time when the Mediterranean Sea did not yet exist. The second occurred during the last 50 Mya, and is noticeable among endemic and native species, but not among exotic species. This period corresponds to isolation of the Mediterranean from Indo-Pacific waters before the Messinian salinity crisis. The Mediterranean fish fauna illustrates well the assembly of regional faunas through origination and immigration, where dispersal and isolation have shaped the emergence of a biodiversity hotspot.

## Introduction

The Mediterranean fish fauna is unique, characterized by a history of isolation and connectivity [1] resulting from tectonic movements and changes in ocean circulation. Isolation of the Mediterranean is reflected in its rich marine flora and fauna, with an estimated total of 17,000 species [1]. 619 fish species have been inventoried in the Mediterranean, among which 13% are endemic, 2% are introduced, and 67% are non-endemic natives. 85% of these fish are teleosts [2]. General geological and oceanographic processes such as those involved at the origin of the Mediterranean Sea have been shown to influence regional histories of fish diversity globally 3,4. Studying the Mediterranean region may therefore illustrate mechanisms contributing to diversification of teleosts and help us understand the current distribution of diversity in the region.

During the Cretaceous (145–65 Mya), the Mediterranean was part of the Tethys Sea and was connected with the Atlantic as well as with the Indo-Pacific oceans. At this time, Africa, Europe and the Adriatic plates were coming closer together, making this ancestral Mediterranean Sea smaller and smaller, and drastically changing its shape and connectivity. By the Miocene (23–5 Mya), the Mediterranean Sea was isolated from the Indo-Pacific. Subsequently, circa 7–5 Mya, it is believed to have been isolated from the Atlantic as well, causing a period of important environmental stress characterised by high desiccation and low sea level known as the Messinian Salinity Crisis (MSC) [5], [6]. During the MSC, the Mediterranean Sea was probably reduced to a series of small lakes, causing a rise in water salinity and a very important extinction crisis among the fish fauna. However, about 5 Mya, the connection with the Atlantic Ocean reopened through the Strait of Gibraltar, allowing colonization of new species into the Mediterranean [5], [6]. Today, the Mediterranean Sea is enclosed by land, with only two small connections to other oceans: the Strait of Gibraltar, and the Suez Canal, an artificial connection to the Red Sea that was opened in 1869 [2]. Despite the Strait of Gibraltar being only 14 km wide, it largely determines water circulation and productivity patterns, especially in the western Mediterranean [7].

A dated phylogeny of teleost taxa specific to the Mediterranean Sea is crucial to understand how episodes of drastic environmental changes in water circulation, environmental conditions, and level of isolation [5] have marked the evolution of its current diversity. To date, however, no phylogenetic reconstruction of teleost fish diversification events in the region has been published. Teleost fish represent the largest vertebrate group on Earth, with an estimated 27,000–31,000 species worldwide [8] (see alsoFishBase, http://www.fishbase.org). Building a phylogeny of teleosts remains challenging and controversial due to the large number of species and the lack of agreement regarding classification of some major orders and families [8], [9]. For example, one of its largest orders, the Perciformes, includes a mixture of fairly disparate polyphyletic taxa [8], [9], [10]. There are published phylogenies for some groups, such as the families Gobiidae [11], Sparidae [12], [13], and Labridae [14], which include several representatives of Mediterranean species. However, the most complete dated teleost phylogeny published to date [15] includes only 16 Mediterranean species and an additional 34 genera (represented by Mediterranean congeners) that occur in the Mediterranean.

The main goal of this study was to reconstruct a dated phylogeny of Mediterranean teleost species based on available molecular data to investigate the potential biogeographic causes that underlie current fish diversity in the Mediterranean Sea. We used the inferred dated phylogeny to explore the possibility that biogeographic events have differentially affected native and exotic species, and to relate major changes in diversity to the Earth history. First, the end-Cretaceous extinction crisis and radiation described for fish at the global scale [3], should be reflected in the Mediterranean for all clades. Second, if the isolation of Atlantic and Indo-Pacific waters was important in the emergence of fish diversity in the Mediterranean Sea, we would expect a peak in clade origin among native species before and until the MSC, at the time when water circulation between these two oceans started to be restricted (∼40–20 Mya). Such a diversification burst would support the idea that limited dispersal from the Atlantic may have played a major role in maintaining and generating biodiversity within the Mediterranean, though we cannot exclude a complementary contribution of other regional mechanisms such as local isolation or extreme environmental conditions. Finally, if allopatric speciation due to the formation of highly isolated lakes during the MSC was the main driver of current diversity, we would expect a more recent origin of native clades centred around the MSC (∼7–5 Mya). In both cases, these peaks should be observed among native and endemic species, but not among exotic species.

## Materials and Methods

### Data harvesting

Nucleotide sequences for Mediterranean teleost fishes (as listed in [16] and references therein), plus 9 additional extra-Mediterranean species were downloaded from GenBank using the seqinr package in R v.2.12.1 [17]. Six loci, each represented by >50 species, were identified for further analyses (Appendix S1 and S2). This minimum taxonomic representation potentially ensured a greater resolving phylogenetic power [18]. The DNA markers selected included 4 mitochondrial genes — 12S ribosomal RNA (12S rDNA; 221 species), 16S ribosomal RNA (16S rDNA; 265 species), cytochrome *c* oxidase subunit I (COXI; 118 species), and cytochrome *b* (CYB; 235 species) —, and two nuclear genes, the intronless rhodopsin (RHO; 183 species) and the recombination activating gene I (RAG1; 80 species). These markers have been used previously to unravel phylogenetic relationships among closely and distantly related species [19], [20], [21], [22], [23], [24], [25]. Because mitochondrial genes display average faster evolutionary rates as compared to nuclear exons, the former provide resolving power for closely related organisms, while the latter provide better resolution for deeper nodes [4], [15].

The final analysis included 363 Mediterranean teleost species (62% of the total number of teleost species in the region), representing all orders, 110 families and 237 genera present in the Mediterranean Sea, and 9 extra-Mediterranean species (see Appendix S1).

### Phylogenetic analyses

Downloaded sequences were individually aligned for each gene using MAFFT [26], version 5. The resulting alignments were inspected and further refined manually. Ambiguous regions of the alignments were filtered using Gblocks [27], version 0.91b. Parameters were set so that the minimum block length was 10 sites, and the maximum number of contiguous non-conserved positions was 5, while conserving sites with a maximum of 50% of gaps. The resulting aligned sequences had the following number of positions (% of the original alignments): 297 (30%) for 12S rRNA, 376 (62%) for 16S rRNA, 622 (58%) for COX1, 1107 (97%) for CYB, 437 (57%) for RHO, and 1,424 (33%) for RAG1. Aligned sequences were then concatenated into a supermatrix of 4,263 sites, and analysed for phylogenetic reconstruction under maximum likelihood (ML) [28]. The best-fitting model of sequence evolution was selected using the Akaike information criterion and hierarchical likelihood ratio tests calculated under Modeltest version 3.7 [29]. Both criteria identified the general time reversible (GTR) model of nucleotide exchangeabilities, with a Gamma (Γ) distribution plus a fraction (I) of invariable sites to account for among-sites substitution rate heterogeneities. All GTR+Γ+I and branch length parameters were estimated from the data.

A preliminary unconstrained analysis resulted in some widely accepted clades being polyphyletic, leading us to enforce the following topological constraints in subsequent tree searches: Clupeiformes + *Danio*, Gadiformes, Lampriformes, Myctophiformes, Pleuronectiformes, Stomiiformes, and Tetraodontiformes for orders [30], [31], [32], [33], and Labridae [14] for families. The orders Scorpaeniformes and Syngnathiformes, and the family Serranidae (Perciformes) were also constrained based on FishBase classification and on the lack of published evidence that these clades would be polyphyletic. Conversely, because there is published evidence that the family *Spicara* (Centracanthidae, Perciformes) is genuinely included within the Sparidae [34], and that the Echeneidae are nested within the Carangidae [22] we did not constrained these taxa. Moreover, we rooted the trees with elopomorphs (here Anguilliformes + Notacanthiformes) as the sister-group of the remaining teleosts.

A first tree was built using the Randomized Accelerated Maximum Likelihood algorithm RAxML [35], v7.0.4. The resulting tree was the starting point for a deeper exploration of the topological space using PAUP* [36], version 4b10. Different cycles of tree search with tree-bisection reconnection (TBR) branch swapping and model parameter re-estimation were performed. The number of TBR rearrangements was increased to 10,000, 50,000, and then 100,000. The search was stopped as no further increase in log-likelihood was observed. The highest-likelihood tree thus identified was taken as the 6-gene best ML phylogenetic hypothesis for subsequent analyses. The corresponding phylograms were subjected to the super-distance matrix (SDM) approach [37] to estimate the relative substitution rate among 12S rDNA, 16S rDNA, COXI, CYB, RHO and RAG1.

Node stability was estimated under ML through 400 replicates of bootstrap re-sampling of the DNA supermatrix [28]. For each replicate, PAUP* computed the highest-likelihood tree based on the re-estimation of the GTR+Γ+I model parameters, with the 6-gene ML topology as a starting point, and 10,000 TBR branch swapping rearrangements. The bootstrap percentages of the consensus tree were mapped on the highest-likelihood phylogram using the bppConsense utility of the Bio++ program suite [38]. All trees were drawn using the APE library [39] within the R statistical package.

### Molecular dating

Divergence times among taxa were estimated using a Bayesian relaxed molecular clock dating strategy [40]. We compiled a list of fossil records and calibrations that have been used in previous publications, and we selected 20 paleontological constraints based on the following criteria:

1. Only primary calibrations were considered, whereas secondary calibrations, based on molecular estimates, were discarded. Following recommendations in [41], minimum and maximum bounds were based solely on fossil information.
2. The fossil record under focus should be unambiguous. For example, a calibration at 161 Mya for Gadiformes [42] is described in [43] as “probable”, though the first certain fossil for this order dates from the Ypresian (56–48 Mya). Because of these discrepancies, we decided to leave this calibration point out.
3. The taxonomic group involved in the calibration should be well resolved in the highest-likelihood phylogeny.

As a result, 20 nodes were constrained according to the available paleontological information (Table 1). For each calibration, we set the minimum (lower) date to the age of the geological stage corresponding to the oldest fossil record. The maximum (upper) bound corresponds to the earliest fossil record for the sister clade, as recommended in [41]. In addition, a 225–152 million years prior was used on the root age for the split between elopomorphs and the remaining teleosts [15]. Due to the incompleteness of the fossil record, all time calibrations were set as soft bounds [44], i.e., 5% of the total probability mass was allocated outside the specified bound. The log-normal rate-autocorrelated model was chosen to relax the molecular clock assumption because of its ability to reasonably fit various data sets [45]. Branch lengths were measured under the CAT mixture model [46], with a general time reversible (GTR) model of exchangeability among nucleotides, and a 4-category Gamma (Γ) distribution of substitution rates across sites to handle different substitution rates among the mitochondrial and nuclear loci. Dating estimates were computed by the Bayesian procedure implemented in the PhyloBayes software [47], version 3.2e (http://www.phylobayes.org). We used the CAT Dirichlet process with the number of components, weights and profiles all inferred from the ML topology, and a birth-death prior on divergence times. Four independent Markov Chains Monte Carlo (MCMC) were run for 4,000 cycles (i.e., 4,000,000 generations), with sampling every 5 cycles. After a burn-in of 200 cycles (i.e., 200,000 generations), log-likelihood and model parameters stabilized. We computed the maximum difference of age estimated for each node by the 4 chains. We observed that the median of these differences in divergence times did not exceed 0.7 Ma, ensuring that convergence had been reached.

**Table 1:** Nodes used for calibration in the phylogeny.

Node Number	Name of clade	Time constraints	Reference
1	Notacanthidae vs Anguilliformes	L94	[43], [69]
2	Anguilliformes	L50	[43]
3	Clupeiformes	L57	[43]
4	Zebrafish vs Medaka (Clupeomorpha)	L150-U165	[70]
5	Myctophidae	L70	[43]
6	Aulopiformes	L96-U128	[15]
7	Tetraodontiformes	L59-U98	[15]
8	Tetraodon vs Takifugu	L32-56	[70]
9	Sparidae	L48	[43]
10	Stickleback vs (Tetraodon+Takifugu)	L97-U151	[70]
11	Gasterosteiformes (stickleback)	L71	[70]
12	*Labrus* vs *Symphodus* *	L40-U84	[43]
13	Gobiidae	L40 – U84	[15]
14	Scombridae	L61	[43]
15	Pleuronectiformes	L51-U99	[15]
16	Soleidae, Pleuronectiformes	L40	[43]
17	Beloniformes	L40	[43]
18	Blenniidae	L40	[43]
19	Pomacentridae	L50 – U84	[15]
20	Medaka vs Stickleback	L97-U151	[70]

* Notice that this node corresponds to the bifurcation between two genera and not to the family Labridae.

**Figure 1:**
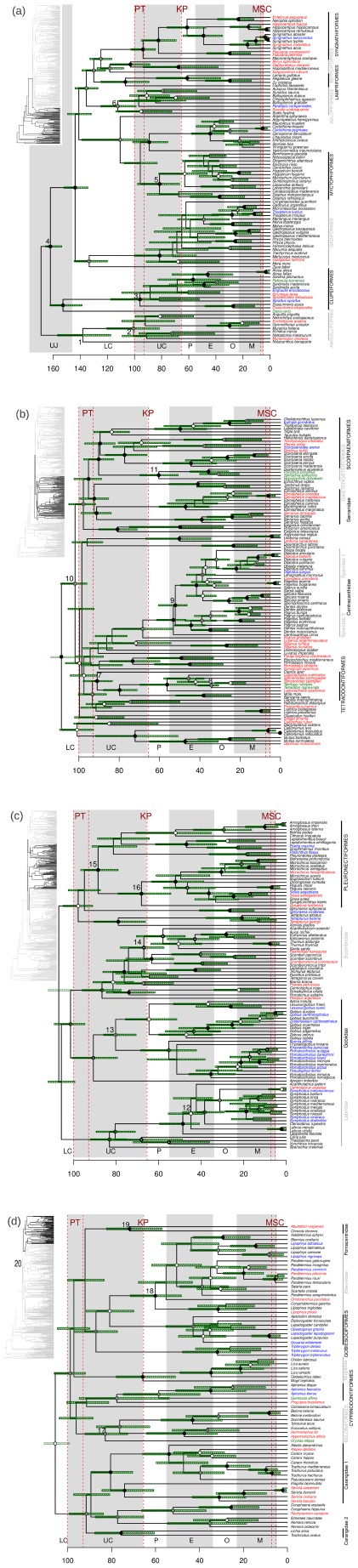
Dated phylogeny of Mediterranean teleost fishes. Phylogenetic relationships and divergence time estimates of Mediterranean teleosts, inferred from a supermatrix of 6 mitochondrial and nuclear genes. The global phylogeny is given on the upper left, with black branches indicating the part of the tree that is represented on the right panel. Color codes on species names indicate the origin of the species: green  =  extra-Mediterranean species; red  =  exotic species; blue  =  endemic species; black  =  non-endemic native. The green dashed boxes around nodes indicate the 95% credibility interval for the estimated node age. Maximum likelihood node bootstrap support is indicated using different types of circles: back circle  =  >90%, double circle  =  70–90%, single white circle  =  50–70%, no circle  = <50%. Letters at the bottom indicate geologic time references: UJ  =  Upper Jurassic; LC  =  Lower Cretaceous; UC  =  Upper Cretaceous; P  =  Paleocene; E  =  Eocene; O  =  Oligocene; M = Miocene. Numbers in the phylogeny correspond to the calibration described in Table 1. Note that node 20 appears on the left panel of Figure 1d as this node links Figure 1b and 1d. We also show with dashed red lines important biogeographic events: peak temperatures (PT) that occurred during the Cenomanian (93–100 Mya); the Cretaceous-Paleogene (KP) mass extinction some 65 Mya, and the Messinian salinity crisis (MSC) some 7–5 Mya. On the right hand side, names in upper case correspond to teleost orders while names in lower case correspond to families. For clarity, names alternate between black and grey.

Node numbers correspond to the numbers shown in Figure 1.

### Diversification events through time

We classified species into natives, exotics and endemics following [16] and references therein: exotic species are species that are found in the Mediterranean Sea and for which there are records of introduction between 1810 and 2006; endemics have a distribution restricted to the Mediterranean; and the rest are non-endemic natives.

We then pruned the full chronogram to study the frequency and timing of diversification events among endemics, non-endemic natives and exotic species by dropping taxa that did not belong to the group of interest (e.g. to build the exotic species tree we dropped all the natives and extra-Mediterranean species). Then we recorded the date of each split and plotted diversification events by time period. To check whether these patterns of diversification were different from random, we sampled the same number of species randomly and without replacement from the full tree (n = 38 for endemics, n = 60 for exotics; n = 263 for non-endemic natives). For each sampling, we recorded the diversification events and calculated their median. This random sampling was repeated 1000 times for each case. We performed a two-tailed significance test i.e. the observed value was considered significantly different from random whenever it was outside the central 95% resampled distribution. We then repeated the same randomizations using either the lower or the upper bounds in the confidence intervals of each node age.

Note that we preferred this randomization strategy over the strategy of estimating diversification rates for each group for two reasons: (1) diversification rates estimates are likely to be biased by incomplete sampling [48] and (2) we are interested here in the timing of diversification events, more than on the estimates of diversification rates that could be obtained using this separate methodology. However we also built lineages-through-time plots for each chronogram analyzed to look at a more precise time-line of diversification events, and compare results from enriched trees (see below).

We finally repeated this diversification analysis increasing the taxonomic coverage. First, we attached to the backbone chronogram additional species for which sequence data were not available but for which congeneric species were already present in the ML topology by “grafting” them as polytomies at the most recent common ancestor (MRCA) [49]. In a first step, we attached species that had 2 congeners represented in the chronogram to the node that linked the two species in question (33 species, see Appendix S3). On a second step, we added species that had at least one congener represented in the chronogram by attaching them to the node that linked the congener with the closest (non-congener) species. Third, we attached species which had members of the same family, and finally those that were represented by members in the same order, attaching them to the node that linked all the members of the same clade. Branch lengths leading to each of the grafted species were set to the age of the node where they had been attached. This resulted in four additional chronograms of increasing taxonomic coverage including 404, 416, 473 and 496 species respectively, the most complete of which includes 93% of all Mediterranean endemics and 87% of non-endemic natives (see Appendix S3 for details).

## Results

### Phylogenetic relationships

While we assumed the monophyly of several groups, many higher level relationships were recovered without the need of imposing constraints on nodes [15], [50], [51]. In this way, Notacanthus is the sister group of Anguilliformes, and Clupeiformes form a deep-branching group, followed by Gadiformes, Myctophiformes, Aulopiformes, and other younger clades (Figure 1a). By contrast, the order Perciformes is polyphyletic, with different families spread along the tree: Sparidae + Centracanthidae, and Serranidae (Figure 1b), Labridae, Gobiidae, and Scombridae (Figure 1c), and Carangidae and Blenniidae (Figure 1d). Three additional families are monophyletic and branch in the crown part of the tree: Mugilidae, Blenniidae, and Pomacentridae (Figure 1d). By contrast, two families are paraphyletic because Echeneidae (*Echeneis* and *Remora*: Figure 1d) is nested within Carangidae [22], and Centracanthidae (Figure 1b) is nested within Sparidae [34]. Within Carangidae, the monophyly of the tribes Carangini and Naucratini [52] is supported.

### Molecular dating

The dated phylogeny suggests that the diversification of the Mediterranean teleosts sampled here started during the Jurassic at least 166–153 Mya (Figure 1a). The diversification of most Perciformes families was estimated to occur during the late Paleocene to mid-Eocene, while the origin of some of the most important orders such as Clupeiformes, Gadiformes and Aulopiformes were dated back to the Cretaceous (Figure 1a). Most large Perciformes families such as Sparidae and Gobiidae started their diversification at around 80–50 Mya (Figure 1b and 1c). Among the youngest Perciformes families we can find Callionymidae, which diversified 14–2 Mya (Figure 1b). Most terminal nodes were dated as <30 Mya, but some exceptions can be found. For example the node that separates the two Pomacentridae species *Abudefduf vaigiensis* and *Chromis chromis* was estimated at 82–57 Mya (Figure 1d).

### Diversification events through time

**Figure 2:**
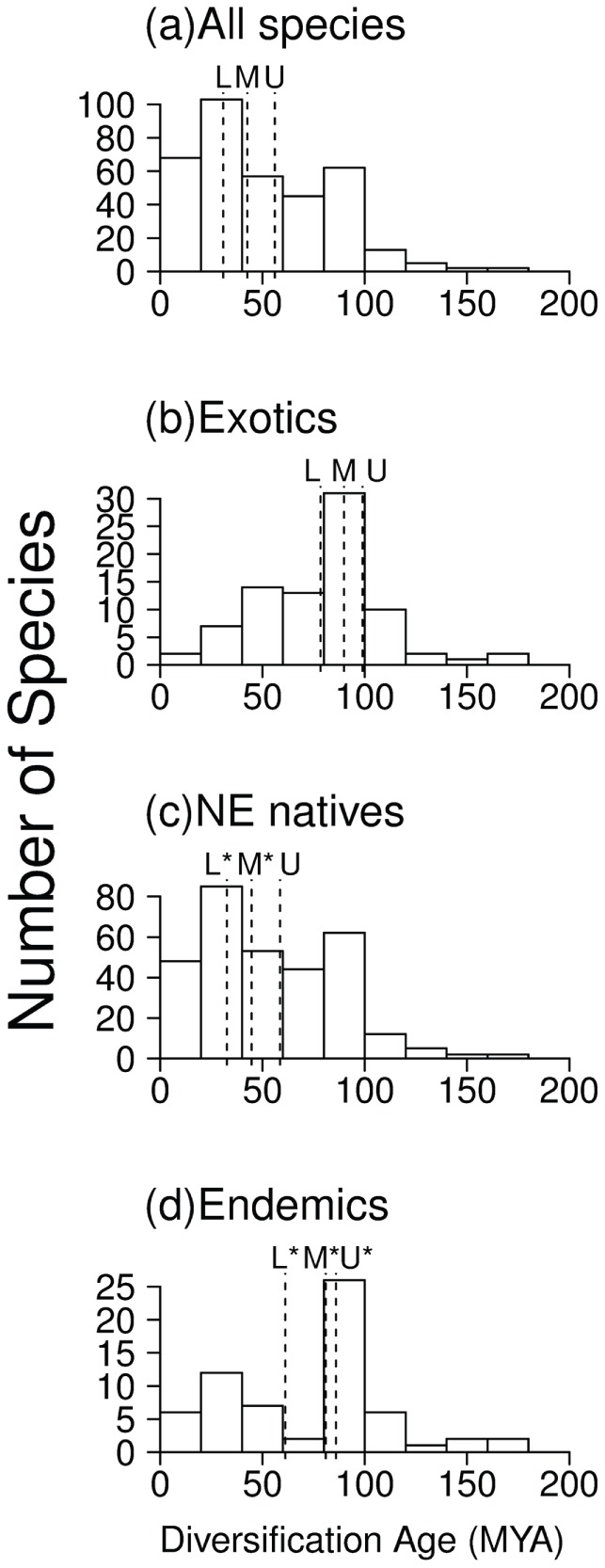
Histograms of the ages of the diversification events. Histograms are shown for (a) all species found in the Mediterranean Sea, (b) exotics only, (c) non-endemic natives, and (d) endemics. Dashed lines indicate the median based on the mean (M) node age estimates as well as based on the lower (L) and upper (U) bounds for each node's 95% credibility interval. Asterisks near the letters indicate significantly different ages than those expected by a random draw of the same number of species from the global chronogram (P<0.05, two-tailed bootstrap test).

**Figure 3:**
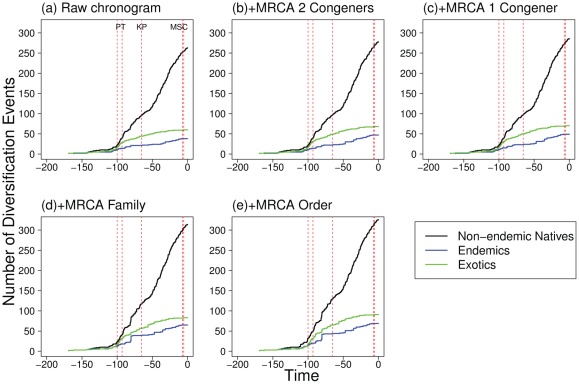
Lineage-through-time plots. Shown for (a) the backbone raw dated phylogeny; (b) adding species represented by at least two congeners in the backbone; (c) adding species represented by at least one congener; (d) adding species represented by a member of its family and (e) adding species represented by a member of its order. We also show with dashed red lines important biogeographic events: PT =  peak temperatures during the Cenomanian (93–100 Mya); KP =  Cretaceous-Paleogene mass extinction some 65 Mya; MSC =  Messinian Salinity Crisis (7–5 Mya).

When all species are considered together in the analysis of diversification, most splitting events took place within the last 40 Mya, with a median at 43 Mya (median at 56–31 Mya if considering upper and lower node age bounds respectively, see Figure 2a). However, this scenario varies when endemics, non-endemic natives and exotic species are considered separately. Exotic species only showed one diversification peak at 100–80 Mya, but no peak during the last 50 Mya (Figure 2b), and presented a median value for diversification events of 90 Mya (99–78 Mya). Non-endemic native species showed a primary peak at 40–20 Mya and a secondary peak at 100–80 Mya (Figure 2c), and had an overall median of 45 Mya (59–33 Mya). Endemics showed a primary peak at 100–80 Mya and a secondary peak at 40–20 Mya (Figure 2d), with an overall median value of 81 Mya (91–70 Mya). Similar diversification patterns are observed when using any of the chronograms enriched by taxon grafting (results not shown), and are supported by the lineage-through-time plots (Figure 3). The slope of the lineage-through-time plots for endemics increases between 100–80 Mya and between 40–10 Mya (Figure 3), as it is observed for non-endemic natives. However, exotic species only showed an increase in the slope after 100 Mya. These patterns remain consistent whether the raw dated phylogeny (Figure 3a), the grafted trees including congeneric representatives (Figure 3b and 3c), or family and order representatives (Figure 3d and 3e) are considered.

To summarize, three general patterns were evidenced in all diversification analyses. First, endemic and native species showed a significantly younger diversification median age than expected by a random draw of the same number of species from the phylogeny (Figure 2). Second, diversification median age of exotic species was not different from random (Figure 2). And finally, natives and endemics showed a peak in diversification in the last 50 Mya that was not found for the exotic pool (Figure 2 and 3).

## Discussion

### Reliability of the teleost phylogeny and timetree estimates

Four elements are crucial to reliably approach the evolutionary history of Mediterranean teleosts in our analysis: taxon sampling, gene sampling, topology inferred, and divergence times. First, we have followed a strategy of increasing taxon sampling at the expense of the number of markers because our focus on the understanding of the diversification patterns of Mediterranean teleosts required a stable phylogenetic picture with a wide taxonomic coverage and a reduced systematic error [53]. Conversely, other studies have favoured the number of genes by comparing complete teleost mitochondrial genomes (e. g. [10]). Second, the relative evolutionary rates among the 6 genes — as measured by the SDM procedure [37] — showed that the slowest-evolving marker is, as expected, the nuclear gene RAG1. Furthermore, the mitochondrial and nuclear DNA supermatrix of ∼4,300 unambiguously aligned sites combined genes with contrasted evolutionary dynamics. This likely provided phylogenetic resolving power at lower taxonomic level for the faster-evolving markers (e.g., CYB, COXI), and at deeper levels for the slower-evolving ones (RAG1, RHO, mitochondrial rDNAs). Certainly the resolution of additional teleost diversification events during intermediate periods of time will require gathering evolutionary signal in complete mitogenomes and other nuclear markers (e.g. [10], [54]). However, considering supplementary genes would have required sequencing *de novo*, which was out of the scope of this project. Third, the amount of missing character states in our supermatrix was 59 %. This reflects our choice of sampling incomplete taxa to maximize the taxonomic coverage. Although this approach may decrease phylogenetic accuracy, it has been shown that the limited availability of complete characters is more important than the excess of missing character states [55]. Therefore, additional taxa involving a non-negligible amount of missing data may not compromise the accuracy of the phylogenetic inference [56]. Fourth, as the phylogenetic tree contains the primary information about both evolutionary rates and divergence times, the estimation of the teleost timetree heavily relies upon the correct measurement of branch lengths through realistic models of sequence evolution. The CAT mixture model used here distributes the alignment sites into categories to handle the site-specific nucleotide preferences [46]. Thanks to its more efficient ability to detect multiple substitutions, branch lengths estimated under the CAT model will be less affected by saturation and will handle the heterogeneity present between nuclear and mitochondrial loci. Finally, we improved the phylogenetic resolution of our tree by securing the monophyly of widely accepted taxa, and leaving other clades unconstrained. Although it can be argued that the constrains impose an additional level of subjectivity in the analysis, as we had to decide which clades needed to be constrained or not, supplementary analyses comparing constrained versus unconstrained trees (results not shown) showed that the timing of speciation events is not influenced by these decisions and that our conclusions are robust to the phylogenetic structure presented here.

### Timeline of the diversification of native and exotic species

Here we draw for the first time a timeline of origin and diversification events for the teleosts of the Mediterranean Sea. Overall, the diversification of all major clades in the Mediterranean (Figure 1) coincides with that published by Santini and colleagues [15] for teleosts at the global scale. Santini *et* *al.* 's work was based on one nuclear gene (RAG1) sampled for 225 species, and 45 calibrations. Here we used more genes to build a dated phylogeny of 372 species, and 20 calibrations. While in [15] species were chosen to maximize the number of teleost orders worldwide, we selected species according to a biogeographic criterion, i.e. their occurrence in the Mediterranean Sea. A major consequence of our strategy was that several orders and families had two or more representatives in the tree, while some others were not represented. Despite these differences in the circumscription of the taxa and phylogenetic markers, all major clades represented in [15] were sampled here. More importantly, the evolutionary history of speciation events in the Mediterranean could not be deduced from a global study such as [15] where only 34 Mediterranean genera and an additional 16 Mediterranean species were represented.

Our results show similar dates of diversification for some of the major orders and families, but they also reveal a difference in tempo between native and exotic species. The fact that median diversification age for exotic species was not different from random, but those of native species was (Figure 2), suggests that speciation within the region has been affected by a succession of biogeographic events at the global but also at the local scale. However, diversification events among native species did not correspond to the MSC, which occurred at around 6 Mya. In fact, natives showed an older diversification peak at 100–80 Mya, and a peak at 40–20 Mya. Lineage-through-time plots (Figure 3) suggest that between these periods of time the number of clades been originated slowed down. Although the incomplete representation of the different taxa may influence our perception of speciation and extinction events, neither lineage-through-time plots (Figure 3) nor comparisons with random expectation (Figure 2) suggest any acceleration of speciation events during the MSC 6 Mya. However they both support two important diversification events for native species (100 Mya and 40 Mya), while only one relevant diversification event for exotics (100 Mya).

According to our diversification estimates, the deepest clades of Mediterranean teleosts would have originated roughly 160 Mya, the Anguilliformes having originated 100 Mya (confidence interval: 120–81 Mya) and the node between Clupeiformes + Danio and the rest of euteleosts been placed at 160 Mya (confidence interval: 166–154 Mya) (Figure 1a). This would place the origin of Mediterranean teleosts shortly after the origin of teleosts globally. Actually, Santini *et al.* [15] found that teleost diversification occurred some 193 Mya (with confidence intervals between 173 and 214 Mya). The first fossil record for teleosts dates back to the upper Jurassic at 151 Mya [4], [57], which is slightly more recent than our molecular estimate for the origin of Mediterranean teleosts. In contrast, some of the molecular calibrations based on mitogenomic data would place the origin of teleosts much earlier, up to 326 Mya [10], . This would imply that Mediterranean teleosts could have a much more recent origin as compared to the global pool, which would also translate into major orders and families originating later. As discussed earlier [4], there is no independent fossil or climatological event that would suggest such an early origin of teleosts. Furthermore, a recent analysis of fish fossil skeletons and otoliths also supports the idea that there is no evidence for such an early origin of teleost fish worldwide [3].

At least two factors may explain the difference in molecular estimates mentioned above [4], [15]. First, previous dating efforts have used a mixed calibration strategy, where the minimum bound was set by the fossil record, whereas the maximum bound incorporated previous molecular estimates. This strategy can have a detrimental effect on the quality of divergence time estimates [59]. Second, studies that have used only mitochondrial genes tend to find older node estimates than those based on nuclear genes [4]. This may be due to differences in substitution rates between nuclear and mitochondrial genes. Here we used a combination of two nuclear and 4 mitochondrial genes analysed under a mixture model to mitigate the relative effects of both types of markers, and we have calibrated our tree based only on fossil records, potentially reducing the pitfalls mentioned above. More importantly, our timeline seems to be in closer agreement with the fossil record and morphological diversification studies [3]. For example, Figure 1 shows that several clades such as Tetraodontiformes, Aulopiformes, Myctophiformes, Clupeiformes and Anguilliformes originated and diversified during the Cretaceous. This is consistent with recent analyses of the fossil record at the global level [3], [60], [61], [62] as well as within the Tethys Sea [60]. In all cases, important radiation events have been observed during the Cretaceous, coinciding with the chronology shown in Figure 1 for the diversification of major fish orders. Such radiations were associated to a global increase in sea surface temperature at the beginning of the Cretaceous, presumably allowing for the evolution and emergence of new clades, followed by the massive extinction at the Cretaceous/Paleogene boundary, which may have triggered the actual diversification and occupation of newly available empty niches [62]. The peak in diversification events after 100 Mya, which can be seen in all fish groups (Figures 2 and 3) is therefore consistent with the idea that high sea level as well as high temperatures during this period of time created new opportunities for speciation and diversification [3], [61], [62]. The fact that native as well as exotic species show this peak (Figure 2) suggests that these global changes that occurred well before the closing of the Mediterranean Sea, had important consequences for the origin of Mediterranean diversity as well. This is also supported by the origination of major Perciformes families during the Paleocene, coinciding with the major morphological diversification of teleosts [3].

Native species originated mostly during the last 50 Mya (Figure 2c–d), by a process that seemed to have started before the MSC. By the beginning of this period the African, Arabic and Eurasian plates were coming closer together, and water flow was slowly stagnating to form what is today the Mediterranean Sea, and effectively separating the Indian and Atlantic oceans. The Eocene-Oligocene transition that corresponds with this period is also marked by large global climate changes. This transition culminated with the MSC at around 6 Mya, which probably eliminated a large portion of fish diversity in the region [5], [6] and where locally surviving species were mostly neritic [63]. However, during this time, the Indian Ocean and the Atlantic fish faunas remained isolated, providing plenty of opportunities for vicariant speciation and promoting a higher diversification rate which has been suggested as the basis of the Mediterranean fish diversity today [5], [6]. Therefore, both the timing of diversification events among natives (Figures 2 and 3) and the analysis of the fossil record in the Mediterranean [63], [64], point to an important role of the separation between the Indian Ocean and the Atlantic Ocean as a driver of current fish diversity in the area. The fossil record also shows a wide variety of fish that are now extinct in the area, suggesting that part of this diversity has been shaped by important extinction events, and a balance between origination and extinction [64]. Adding to this evidence, paleontological analyses in the Meditarranean have already demonstrated that the picture regarding the MSC is not as simple as originally thought, i.e. that the Mediterranean was not hyper saline everywhere and that many species could have survived extinction locally [63]. In particular, biochemical analysis of sediments and faunal fossils including otoliths have shown that some interior parts of the Mediterranean, specifically in Italy, would have been connected to the Sea and would have shown salinity levels comparable to those currently present in the Mediterranean Sea [64], [65], [66]. Therefore, the MSC may have played a rather secondary role in speciation events leading to the current fish diversity in the Mediterranean.

Certainly our results regarding the tempo of diversification could have been influenced by our coverage of the different groups analysed. For example, in the raw dated phylogeny we represented 46% of all endemic teleosts in the Mediterranean (Figure 3a). Attaching species to the most recent common ancestors if they had at least one congener represented increases this representation to 66% (Figure 3c, see Appendix S3 for number of species added at each level). Finally, by also considering species that had a member of the same family (Figure 3d) or on the same order (Figure 3e) we increased the coverage of endemics to 92%. Although one may argue that the patterns observed in the most complete chronogram are due to an artefact of adding species to deeper family and order nodes, this argument cannot be applied to the analysis carried out adding only congeners to the backbone tree. Here, one would expect accentuated patterns that are already present in the backbone chronogram for endemic species. These analyses do not show any increase in diversification of endemics or natives during the MSC, but they always show the two above-mentioned peaks after 100 Mya and 40 Mya (Figure 3). Therefore we expect that these patterns will be robust to analyses using further gene sequencing and additional species.

### Conclusions

Overall our results show that fish diversity in the Mediterranean Sea originated largely during the Cretaceous and Paleocene during episodes of global change, when the Mediterranean Sea still did not exist. They also suggest that the isolation between Atlantic and Indo-Pacific waters before the MSC had a large role in the emergence of native and endemic species diversity. Beyond the establishment of phylogenetic relationships among Mediterranean marine fish and advances in the comprehension of evolutionary history underlying this diversity, our study paves the way towards a phylogenetic perspective in the conservation of fish biodiversity at a macroecological scale [67]. In a different vein, understanding the interplay between phylogenetic diversity and environmental gradients at large biogeographic scales may also help us understand the mechanisms that are behind the emergence and maintenance of diversity [68]. This understanding is fundamental in the Mediterranean Sea where biodiversity may be at high risk under the rates of current global changes [1], [2], [6], [67].

## Supporting Information

Appendix S1 **Catalog of GenBank sequences used in the phylogenetic analysis.**
(DOC)

Appendix S2 **Gene representation and saturation in the phylogenetic analysis.**
(DOC)

Appendix S3 **Species grafted at their most recent common ancestor** (**MRCA**)**.**
(DOC)
