# Supplementary material for: A Phylogenetic Perspective on the Evolution of Mediterranean Teleost Fishes
Source: PLoS One. 2012 May 8;7(5):e36443. doi: 10.1371/journal.pone.0036443 (PMC3348158; doi:10.1371/journal.pone.0036443)
Supplement: Appendix S1 — Catalog of GenBank sequences used in the phylogenetic analysis. (DOC) [file pone.0036443.s001.doc]

**Appendix Table S1**: **GenBank sequence catalog.**

| **ORDER** | **FAMILY** | **SPECIES** | **12S** | **16S** | **COXI** | **CytB** | **Rhod** | **RAG1** |
| --- | --- | --- | --- | --- | --- | --- | --- | --- |
| Anguilliformes | Anguillidae | *Anguilla anguilla* (Linnaeus 1758) | AF266494 | AB021749 | AP007233 | AB021776 | L78007 |  |
|  | Muraenesocidae | *Muraenesox cinereus* (Forsskål 1775) | AF417318 |  | EF607449 | AY295080 |  |  |
|  | Muraenidae | *Enchelycore anatina* (Lowe 1838) |  |  |  | EF439251 | EF427475 |  |
|  |  | *Gymnothorax unicolor* (Delarohe 1809) |  |  |  |  | AY862128 |  |
|  |  | *Muraena helena* Linnaeus 1758 |  |  |  | AY862092 | AY862118 |  |
|  | Nemichthyidae | *Nemichthys scolopaceus* Richardson 1848 | AB049989 | AY952481 | EU148262 | AB038418 |  |  |
|  | Nettastomatidae | *Nettastoma melanurum* Rafinesque 1810 | DQ645673 | DQ645712 |  |  |  |  |
|  | Ophichthidae | *Echelus myrus* (Linnaeus 1758) | DQ645651 | DQ645690 |  |  |  |  |
| Aulopiformes | Aulopidae | *Aulopus filamentosus* (Bloch 1792) |  |  |  |  | EF439259 | EU366688 |
|  | Chlorophthalmidae | *Chlorophthalmus agassizi* Bonaparte 1840 | AP002918 | DQ027906 | DQ027975 | EF439508 | EF439358 | FJ896455 |
|  | Ipnopidae | *Bathypterois dubius* Vaillant 1888 | AY141326 | AY141396 |  |  | AY141257 |  |
|  |  | *Bathypterois grallator* (Goode & Bean 1886) |  |  |  |  |  | EU366690 |
|  | Paralepididae | *Paralepis coregonoides* Risso 1820 |  |  |  |  |  | EU366708 |
|  |  | *Sudis hyalina* Rafinesque 1810 | EU574933 |  |  |  |  |  |
|  | Synodontidae | *Saurida undosquamis* (Richardson 1848) | AP002920 | AB297971 | AP002920 | AP002920 |  | EU366712 |
|  |  | *Synodus saurus* (Linnaeus 1758) | AF049723 | AF049733 |  | DQ198009 | DQ197911 |  |
| Batrachoidiformes | Batrachoididae | *Halobatrachus didactylus* (Bloch & Schneider 1801) |  | AY368308 |  | AF165351 | AY368323 |  |
| Beloniformes | Adrianichthyidae | *Oryzias latipes* (Temminck & Schlegel 1846) | AP008948.1 | AP008948.1 | AP008948.1 | AP008948.1 | NM_001104695.1 | EF095641 |
|  | Belonidae | *Belone belone gracilis* (Linnaeus 1761) | AF231541 | AF231514 |  | EU036423 | AY141268 |  |
|  |  | *Belone svetovidovi* Collette & Parin 1970 |  | AF243956 |  | AF243880 |  |  |
|  |  | *Tylosurus acus* (Lacepède 1803) | AF231571 | AF231528 |  | AF231656 | EF427530 |  |
|  | Exocoetidae | *Exocoetus volitans* Linnaeus 1758 | AP002933 | AP002933 | AP002933 | AP002933 |  |  |
|  | Hemiramphidae | *Hemiramphus far* (Forsskål 1775) |  | AY693487 | EU148546 | AY693516 |  |  |
|  |  | *Hyporhamphus affinis* (Günther 1866) |  |  | EF609376 |  |  |  |
|  | Scomberesocidae | *Scomberesox saurus* (Walbaum 1792) |  | AF243984 |  | AB355963 |  | AY308771 |
| Beryciformes | Berycidae | *Beryx splendens* Lowe 1834 | AF092197 | AF100909 | EF609297 | AB108491 | AY141265 | EF095636 |
|  | Holocentridae | *Sargocentron rubrum* (Forsskål 1775) | AP004432 | AP004432 | AP004432 | AP004432 |  |  |
|  | Trachichthyidae | *Gephyroberyx darwinii* (Johnson 1866) |  |  | DQ108100 |  |  |  |
|  |  | *Hoplostethus mediterraneus* Cuvier 1829 | AY141335 |  | DQ885093 |  | AY141264 | EF095635 |
| Clupeiformes | Clupeidae | *Alosa alosa* (Linnaeus 1758) | AP009131 | AP009131 | AP009131 | EU224046 | EU224142 |  |
|  |  | *Alosa fallax* (Lacepède, 1803) |  | EU552737 |  | EU552574 | EU491985 |  |
|  |  | *Dussumieria acuta* Valenciennes 1847 |  |  | EU014222 |  |  |  |
|  |  | *Dussumieria elopsoides* Bleeker 1849 |  | EU364556 | EF607361 |  |  |  |
|  |  | *Etrumeus teres* (DeKay 1842) | DQ912038 | DQ912073 | AP009139 | EU552621 |  | DQ912110 |
|  |  | *Pellonula leonensis* Boulenger 1916 | NC_009591.1 | NC_009591.1 | NC_009591.1 | NC_009591.1 |  | DQ912130 |
|  |  | *Sardina pilchardus* (Walbaum 1792) | DQ912053 | DQ912088 | EF609451 | AF472582 | EF439304 |  |
|  |  | *Sardinella aurita* Valenciennes 1847 | DQ912032 | DQ912067 | AM911173 | EU552619 | EF439427 | DQ912104 |
|  |  | *Sardinella maderensis* (Lowe 1838) | AP009143 | AM911205 | AM911175 | AF472583 | EF439303 |  |
|  |  | *Spratelloides delicatulus* (Bennett 1832) | DQ912058 | DQ912093 | AP009144 | AP009144 |  | DQ912128 |
|  |  | *Sprattus sprattus* (Linnaeus 1758) | AP009234 | AM911201 | AM911177 | AF472581 | EU491991 |  |
|  | Engraulidae | *Engraulis encrasicolus* (Linnaeus 1758) | DQ912031 | DQ912066 | AM911182 | EU552563 | EU224151 | DQ912103.1 |
| Cypriniformes | Cyprinidae | *Danio rerio* (Hamilton 1822) | NC_002333.2 | NC_002333.2 | NC_002333.2 | NC_002333.2 | NM_131084.1 | U71093 |
| Cyprinodontiformes | Cyprinodontidae | *Aphanius dispar* (Rüppell 1829) |  | ADU05964 |  |  |  |  |
|  |  | *Aphanius fasciatus* (Valenciennes 1821) |  | AFU05965 |  | AF299273 |  |  |
|  |  | *Aphanius iberus* (Valenciennes 1846) |  |  |  | AF299274 |  |  |
|  | Poesiliidae | *Gambusia affinis* (Baird & Girard 1853) | NC_004388.1 | NC_004388.1 | NC_004388.1 | NC_004388.1 |  |  |
| Dactylopteriformes | Dactylopteridae | *Dactylopterus volitans* (Linnaeus 1758) | AF150006 |  |  | EF439514 | AY141282 |  |
| Gadiformes | Gadidae | *Gadiculus argenteus* Guichenot 1850 |  |  |  | EU224053 | EU224201 |  |
|  |  | *Merlangius merlangus* (Linnaeus 1758) | AY141330 |  | EF609406 | DQ174057 | AY141260 | FJ215265 |
|  |  | *Micromesistius poutassou* (Risso 1827) | AY842451 | AY850366 |  | DQ174068 | EU492215 |  |
|  |  | *Trisopterus luscus* (Linnaeus 1758) |  |  | EF609486 | DQ174081 | EU224138 |  |
|  |  | *Trisopterus minutus* (Linnaeus 1758) | AY845395 | AY862158 |  | DQ174083 | EU036622 |  |
|  | Lotidae | *Gaidropsarus biscayensis* (Collett 1890) |  |  |  | EF427562 | EF439115 |  |
|  |  | *Gaidropsarus mediterraneus* (Linnaeus 1758) |  |  |  | EF427563 | EF439117 |  |
|  |  | *Gaidropsarus vulgaris* (Cloquet 1824) |  |  |  | DQ174050 |  |  |
|  |  | *Molva dypterygia* (Pennant 1784) |  |  |  | AF469625 | EF439140 |  |
|  |  | *Molva molva* (Linnaeus 1758) |  |  |  | DQ174071 | EF439141 | FJ215275 |
|  | Macrouridae | *Coryphaenoides guentheri* (Vaillant 1888) |  |  | EU148125 |  |  |  |
|  |  | *Hymenocephalus italicus* Giglioli 1884 |  |  |  |  |  | FJ215246 |
|  |  | *Nezumia aequalis* (Günther 1878) |  |  |  |  |  | FJ215280 |
|  |  | *Trachyrincus scabrus* (Rafinesque 1810) |  |  |  |  |  | FJ215298 |
|  | Merlucciidae | *Merluccius merluccius* (Linnaeus 1758) | DQ274008 |  | EF609408 | DQ174062 | EF439400 |  |
|  | Moridae | *Guttigadus latifrons* (Holt & Byrne 1908) |  |  | EU148219 |  |  |  |
|  |  | *Mora moro* (Risso 1810) | AY368285 | AY368307 | EF609410 | DQ197964 | AY368322 |  |
|  | Phycidae | *Phycis blennoides* (Brünnich 1768) | AY845393 | AY850365 |  | DQ174072 | AY368321 |  |
|  |  | *Phycis phycis* (Linnaeus 1766) |  |  |  | DQ197978 | DQ197880 |  |
| Gasterosteiformes | Gasterosteidae | *Gasterosteus aculeatus* Linnaeus 1758 | NC_003174.1 | NC_003174.1 | NC_003174.1 | NC_003174.1 | EU637962.1 | EF033039 |
|  |  | *Pungitius pungitius* (Linnaeus 1758) | NC_011571.1 | NC_011571.1 | NC_011571.1 | NC_011571.1 |  | AB445183 |
|  |  | *Spinachia spinachia* (Linnaeus 1758) | NC_011582.1 | NC_011582.1 | NC_011582.1 | NC_011582.1 | AY141281.1 | AB445184 |
|  | Hypoptychidae | *Hypoptychus dybowskii* Steindachner 1880 | NC_004400 | NC_004400 | NC_004400 | NC_004400 |  | AB445176 |
| Gobiesociformes | Gobiesocidae | *Apletodon dentatus* (Facciolà 1887) | AF549200 | AF549207 |  |  | AY141274 |  |
|  |  | *Diplecogaster bimaculata* (Bonnaterre 1788) | AF549197 | AF549205 |  |  |  |  |
|  |  | *Gouania willdenowi* (Risso 1810) | EF363030 | EF363032 |  |  |  |  |
|  |  | *Lepadogaster candollei* Risso 1810 | AY036588 | AF549203 |  |  |  |  |
|  |  | *Lepadogaster lepadogaster* (Bonnaterre 1788) | AY036589 | AF549202 |  |  | AY141273 |  |
|  |  | *Lepadogaster purpurea* (Bonnaterre 1788) | AY036599 | AF549201 |  |  |  |  |
|  |  | *Opeatogenys gracilis* (Canestrini 1864) | AF549196 | AF549206 |  |  |  |  |
| Lampriformes | Lampridae | *Lampris guttatus* (Brünnich 1788) | AF049726 | AF049736 | DQ885096 | DQ197959 |  | AY308764 |
|  | Lophotidae | *Lophotus lacepede* Giorna 1809 | AY036616 | AY036618 |  |  |  | FJ896461 |
|  | Regalecidae | *Regalecus glesne* Ascanius 1772 | AF049728 | EU099465 |  |  | AY368328 | EF107625 |
|  | Trachipteridae | *Zu cristatus* (Bonelli 1819) | AY652748 | AY652749 |  |  |  | FJ896462 |
| Lophiiformes | Lophiidae | *Lophius budegassa* Spinola 1807 | EF095552 |  |  | EF427574 | EF095608 | EF095637 |
|  |  | *Lophius piscatorius* Linnaeus 1758 | AY368294 |  |  | EF427575 | AY368325 |  |
| Myctophiformes | Myctophidae | *Benthosema glaciale* (Reinhardt 1837) |  | DQ532843 | EU148098 |  |  | EU366728 |
|  |  | *Ceratoscopelus maderensis* (Lowe 1839) |  |  | EU148109 |  |  |  |
|  |  | *Diaphus metopoclampus* (Cocco 1829) |  |  | EU148149 |  |  |  |
|  |  | *Diaphus rafinesquii* (Cocco 1838) |  |  | EU148152 |  |  |  |
|  |  | *Diogenichthys atlanticus* (Tåning 1928) |  | AB042178 |  |  |  |  |
|  |  | *Electrona risso* (Cocco 1829) |  |  | EU148157 |  |  |  |
|  |  | *Gonichthys cocco* (Cocco 1829) |  |  | EU148175 |  |  |  |
|  |  | *Hygophum benoiti* (Cocco 1838) |  | AB024912 | EU148202 |  |  |  |
|  |  | *Hygophum hygomii* (Lütken 1892) | AF049724 | AB024915 | EU148205 |  |  | EF094947 |
|  |  | *Lobianchia dofleini* (Zugmayer 1911) |  | DQ532898 |  |  |  |  |
|  |  | *Lobianchia gemellarii* Cocco 1838 |  | AB042159 |  |  |  |  |
|  |  | *Myctophum punctatum* Rafinesque 1810 |  | AF221864 | EU148251 |  |  |  |
|  |  | *Notoscopelus bolini* Nafpaktitis 1975 |  |  | EU148276 |  |  |  |
|  |  | *Symbolophorus veranyi* (Moreau 1888) |  |  | EU148340 |  |  |  |
| Notacanthiformes | Notacanthidae | *Notacanthus bonaparte* Risso 1840 | X99182 | X99181 | EU148274 |  |  |  |
| Osmeriformes | Argentinidae | *Argentina sphyraena* Linnaeus 1758 |  |  |  | EU492324 | EU492231 |  |
| Perciformes | Acropomatidae | *Synagrops japonicus* (Döderlein 1883) |  | EF120861 |  | AB104919 |  |  |
|  | Apogonidae | *Apogon imberbis* (Linnaeus 1758) | AM158282 | FJ462721 |  |  |  |  |
|  | Blenniidae | *Aidablennius sphynx* (Valenciennes 1836) | AF549191 | AF549193 |  |  |  |  |
|  |  | *Blennius ocellaris* Linnaeus 1758 | AY098746 | AY098815 |  |  |  |  |
|  |  | *Coryphoblennius galerita* (Linnaeus 1758) | AY098748 | AY098816 |  |  |  |  |
|  |  | *Lipophrys adriaticus* (Steindachner & Kolombatovic 1883) | AY098758 | AF324188 |  |  |  |  |
|  |  | *Lipophrys canevae* (Vinciguerra 1880) | AF414713 | AY098821 |  |  |  |  |
|  |  | *Lipophrys dalmatinus* (Steindachner & Kolombatovic 1883) | AY098756 | AY098823 |  |  |  |  |
|  |  | *Lipophrys nigriceps* (Vinciguerra 1883) | AF414714 | AY098824 |  |  |  |  |
|  |  | *Lipophrys pholis* (Linnaeus 1758) | AY098761 | AY098825 |  |  |  |  |
|  |  | *Lipophrys trigloides* (Valenciennes 1836) | AY098768 | AY987024 |  |  |  |  |
|  |  | *Omobranchus punctatus* (Valenciennes 1836) | OPU90393 |  |  |  |  |  |
|  |  | *Parablennius gattorugine* (Linnaeus 1758) | AF414715 | DQ160198 |  |  | AY141271 |  |
|  |  | *Parablennius incognitus* (Bath 1968) | AY098784 | AY098829 |  |  |  |  |
|  |  | *Parablennius pilicornis* (Cuvier 1829) | AY098795 | AY098831 |  |  |  |  |
|  |  | *Parablennius rouxi* (Cocco 1833) | AY098781 | AY098833 | AJ872148 |  |  |  |
|  |  | *Parablennius sanguinolentus* (Pallas 1814) | AF414697 | AF428241 |  |  |  |  |
|  |  | *Parablennius tentacularis* (Brünnich 1768) | AY098780 | AY098838 |  |  |  |  |
|  |  | *Parablennius zvonimiri* (Kolombatovic 1892) | AY098790 | AY098840 |  |  |  |  |
|  |  | *Salaria pavo* (Risso 1810) | AY098798 | AY098842 |  |  |  |  |
|  |  | *Scartella cristata* (Linnaeus 1758) | AY098803 | AY098845 |  |  |  |  |
|  | Bramidae | *Brama brama* (Bonnaterre 1788) |  |  | EF609300 | DQ197933 | DQ197835 |  |
|  | Callanthiidae | *Callanthias ruber* (Rafinesque 1810) |  | EF120863 |  |  | EU637945 |  |
|  | Callionymidae | *Callionymus lyra* Linnaeus 1758 | AY141344 | AY141414 |  |  | AY141270 |  |
|  |  | *Callionymus maculatus* Linnaeus 1810 |  |  |  |  | EU491964 |  |
|  |  | *Callionymus reticulatus* Valenciennes 1837 |  |  |  |  | EU491962 |  |
|  | Carangidae | *Alectis alexandrinus* (Geoffroy Saint-Hilaire 1817) |  |  |  | AF363738 |  | EU167759 |
|  |  | *Alepes djedaba* (Forsskål 1775) |  | EF613269 | EF607307 | EF512295 |  |  |
|  |  | *Caranx crysos* (Mitchill 1815) |  |  |  | AY050717 | EF427461 |  |
|  |  | *Caranx hippos* (Linnaeus 1766) |  | DQ532847 |  | AY050720 |  | EU167758 |
|  |  | *Caranx rhonchus* Geoffroy Saint-Hilaire 1817 |  |  |  | AY050733 |  |  |
|  |  | *Elagatis bipinnulata* (Quoy & Gaimard 1825) |  |  | EU014213 | AY050734 |  |  |
|  |  | *Lichia amia* (Linnaeus 1758) |  |  |  | EF392593 | EF427481 |  |
|  |  | *Pseudocaranx dentex* (Bloch & Schneider 1801) |  |  | EF609442 | EF392607.1 | EF439301 |  |
|  |  | *Seriola carpenteri* Mather 1971 |  |  |  | EF392617 | EF427512 |  |
|  |  | *Seriola dumerili* (Risso 1810) |  |  | EF607552 | AB292794 | EF439446 |  |
|  |  | *Seriola fasciata* (Bloch 1793) |  |  |  | AY050748 | EF439317 |  |
|  |  | *Seriola rivoliana* Valenciennes 1833 |  |  |  | AB264297 | EF427516 |  |
|  |  | *Trachinotus ovatus* (Linnaeus 1758) | AY141388 | DQ027921 | DQ027991 | AY050750 | AY141314 |  |
|  |  | *Trachurus mediterraneus* (Steindachner 1868) |  | AF487412 |  | AY526548 | EU036619 |  |
|  |  | *Trachurus picturatus* (Bowdich 1825) |  | AF487415 | EU148351 | AY526546 | EF439329 |  |
|  |  | *Trachurus trachurus* (Linnaeus 1758) | AB108498 | AF487410 | AB108498 | AY526533 | EU491981 |  |
|  | Centracanthidae | *Centracanthus cirrus* Rafinesque 1810 |  |  |  |  |  | EU167766 |
|  |  | *Spicara flexuosa* Rafinesque 1810 |  |  |  | EU036502 | EU036606 | EU167804 |
|  |  | *Spicara maena* (Linnaeus 1758) | AP009164 | AF247434 | AP009164 | AF240737 | EU036610 | EU167805 |
|  |  | *Spicara smaris* (Linnaeus 1758) |  |  |  | EF439599 | EF439465 |  |
|  | Centrolophidae | *Centrolophus niger* (Gmelin 1789) |  | AB205412 | AB205434 | AB205456 | EF439348 |  |
|  |  | *Schedophilus ovalis* (Cuvier 1833) |  | AB205413 | AB205435 | AB205457 | EF427506 |  |
|  | Cepolidae | *Cepola macrophthalma* (Linnaeus 1758) |  | DQ027923 | DQ027993 |  | EF439350 | EU167817 |
|  | Chaetodontidae | *Chaetodon hoefleri* Steindachner 1881 | EF616824 | EF616908 |  |  |  |  |
|  | Cichlidae | *Cichlasoma bimaculatum* (Linnaeus 1758) |  | EF432874 | AY263863 | AF145128 |  | EU706368 |
|  | Coryphaenidae | *Coryphaena equiselis* Linnaeus 1758 |  |  |  | DQ080244 | DQ080342 |  |
|  |  | *Coryphaena hippurus* Linnaeus 1758 | DQ874715 | AY857955 | DQ885087 | AY050761 | DQ874824 | EU167822 |
|  | Echeneidae | *Echeneis naucrates* Linnaeus 1758 | AY141389 | DQ532869 |  | AY050763 | AY141315 | EU167829 |
|  |  | *Remora osteochir* (Cuvier 1829) | EU574934 |  |  |  |  |  |
|  |  | *Remora remora* (Linnaeus 1758) |  | AY836584 | EU403077 |  |  |  |
|  | Epigonidae | *Epigonus constanciae* (Giglioli 1880) |  | EF120867 |  |  |  |  |
|  |  | *Epigonus telescopus* (Risso 1810) |  |  | EF609350 | DQ197949 | DQ197851 | EU167904 |
|  | Gempylidae | *Ruvettus pretiosus* Cocco 1833 | EU003538 | DQ874736 | EU003556 | DQ080265 | DQ874813 |  |
|  | Gobiidae | *Aphia minuta* (Risso 1810) | EF218623 | EF218638 |  |  |  |  |
|  |  | *Buenia affinis* Iljin 1930 | EF218628 | EF218643 |  |  |  |  |
|  |  | *Crystallogobius linearis* (Düben 1845) | EF218635 | EF218650 |  |  |  |  |
|  |  | *Gobius auratus* Risso 1810 | AF067254 | AF067267 |  |  |  |  |
|  |  | *Gobius bucchichi* Steindachner 1870 | EF218627 | EF218642 |  |  |  |  |
|  |  | *Gobius cobitis* Pallas 1814 | EF218629 | EF218644 |  |  |  |  |
|  |  | *Gobius cruentatus* Gmelin 1789 | EF218626 | EF218641 |  |  |  |  |
|  |  | *Gobius niger* Linnaeus 1758 | EF218630 | EF218645 |  | AY884591 |  |  |
|  |  | *Gobius paganellus* Linnaeus 1758 | EF218636 | AF518216 |  |  |  |  |
|  |  | *Gobius xanthocephalus* Heymer & Zander 1992 | DQ382237 |  |  |  |  |  |
|  |  | *Knipowitschia panizzae* (Verga 1841) | AF067259 | AJ616812 |  |  |  |  |
|  |  | *Lesueurigobius friesii* (Malm 1874) | EF218624 | EF218639 |  |  |  |  |
|  |  | *Lesueurigobius suerii* (Risso 1810) | EF218625 | EF218640 |  |  |  |  |
|  |  | *Pomatoschistus canestrinii* (Ninni 1883) | AJ616818 | AJ616835 |  |  |  |  |
|  |  | *Pomatoschistus knerii* (Steindachner 1861) | EF218632 | EF218647 |  |  |  |  |
|  |  | *Pomatoschistus marmoratus* (Risso 1810) | AF067262 | AF067275 |  |  |  |  |
|  |  | *Pomatoschistus microps* (Krøyer 1838) | AJ616811 | AJ616828 |  | AJ550471 |  |  |
|  |  | *Pomatoschistus minutus* (Pallas 1770) | EF218633 | EF218648 |  | AY940726 |  |  |
|  |  | *Pomatoschistus norvegicus* (Collett 1902)  *)* | AJ616814 | AJ616831 |  |  |  |  |
|  |  | *Pomatoschistus pictus* (Malm 1865) | AJ616807 | AJ616834 |  |  |  |  |
|  |  | *Pomatoschistus quagga* (Heckel 1837) | AF067264 | AF067277 |  |  |  |  |
|  |  | *Pseudaphya ferreri* (de Buen & Fage 1908) | EF218631 | EF218646 |  |  |  |  |
|  |  | *Zebrus zebrus* (Risso 1827) | AF067266 | AF067279 |  |  |  |  |
|  |  | *Zosterisessor ophiocephalus* (Pallas 1814) | EF218634 | EF218649 |  | AY884592 |  |  |
|  | Haemulidae | *Parapristipoma octolineatum* (Valenciennes 1833) |  |  |  | DQ197977 | DQ197879 | HQ676666 |
|  |  | *Plectorhinchus mediterraneus* (Guichenot 1850) |  |  |  | DQ197979 | DQ197881 |  |
|  |  | *Pomadasys incisus* (Bowdich 1825) |  | EU410417 |  | DQ197981 | DQ197883 | HQ676679 |
|  |  | *Pomadasys stridens* (Forsskål 1775) |  |  |  |  |  | HQ676685 |
|  | Istiophoridae | *Tetrapturus albidus* Poey 1860 | DQ854632 |  |  | DQ882009 |  |  |
|  |  | *Tetrapturus belone* Rafinesque 1810 | DQ854640 |  |  | DQ882010 |  |  |
|  |  | *Tetrapturus georgii* Lowe 1841 | DQ854642 |  |  | DQ882011 |  |  |
|  | Labridae | *Acantholabrus palloni* (Risso 1810) |  | AF517587 |  | DQ197923 | DQ197825 |  |
|  |  | *Centrolabrus exoletus* (Linnaeus 1758) | AF414200 | AY092041 |  |  |  |  |
|  |  | *Coris julis* (Linnaeus 1758) | AJ810130 | AY092042 |  | AY328856 |  | EU167885 |
|  |  | *Ctenolabrus rupestris* (Linnaeus 1758) | AJ810131 | AF517586 |  |  |  |  |
|  |  | *Labrus merula* Linnaeus 1758 | AJ810141 | AF517592 |  |  |  |  |
|  |  | *Labrus viridis* Linnaeus 1758 | AJ810142 | AF517593 |  |  |  |  |
|  |  | *Lappanella fasciata* (Cocco 1833) |  | AF517589 |  |  |  |  |
|  |  | *Symphodus bailloni* (Valenciennes 1839) | AY092052 | AY092037 |  |  |  |  |
|  |  | *Symphodus cinereus* (Bonnaterre 1788) | AJ810147 | AY092036 |  |  |  |  |
|  |  | *Symphodus doderleini* Jordan 1890 |  | AF517602 |  |  |  |  |
|  |  | *Symphodus mediterraneus* (Linnaeus 1758) | AJ810148 | AF517601 |  |  |  |  |
|  |  | *Symphodus melanocercus* (Risso 1810) | AJ810149 | AF517595 |  |  |  |  |
|  |  | *Symphodus melops* (Linnaeus 1758) | AF414197 | AY092038 |  |  |  |  |
|  |  | *Symphodus ocellatus* (Linnaeus 1758) | AJ810150 | AF517603 |  |  |  |  |
|  |  | *Symphodus roissali* (Risso 1810) | AJ810151 | AY092039 |  |  |  |  |
|  |  | *Symphodus rostratus* (Bloch 1791) | AF414198 | AY092040 |  |  |  |  |
|  |  | *Symphodus tinca* (Linnaeus 1758) | AJ810152 | AF517596 |  |  |  |  |
|  |  | *Thalassoma pavo* (Linnaeus 1758) |  |  |  | AY328877.1 | DQ197913 |  |
|  |  | *Xyrichtys novacula* (Linnaeus 1758) |  |  |  | EF439246 | EF439331 |  |
|  | Lutjanidae | *Lutjanus argentimaculatus* (Forsskål 1775) | AY484978 | DQ444481 | DQ885104 | DQ900672 |  | EU627659 |
|  | Luvaridae | *Luvarus imperialis* Rafinesque 1810 | AY057234 | AY264587 | AP009161 | AB276966 |  | EF530099 |
|  | Moronidae | *Dicentrarchus labrax* (Linnaeus 1758) | AY141370 | AY141440 |  | EF427553 | EU492059 | AH008179 |
|  |  | *Dicentrarchus punctatus* (Bloch 1792) |  | AF247437 |  | AF143191 | DQ197846 |  |
|  | Mugilidae | *Chelon labrosus* (Risso 1827) | DQ016292 | AY169697 |  | DQ197935 | DQ197837 |  |
|  |  | *Liza aurata* (Risso 1810) | EF437077 | AY169698 |  | EF427572 | EF439127 |  |
|  |  | *Liza ramado* (Risso 1827) | EF437079 | AY169700 |  | EU224058 | EU224158 |  |
|  |  | *Liza saliens* (Risso 1810) | EF437081 | AY169702 |  | Z70774 |  |  |
|  |  | *Mugil cephalus* Linnaeus 1758 | DQ225772 | DQ307686 | EF607446 | DQ225777 | EF095609 | EF095639 |
|  |  | *Oedalechilus labeo* (Cuvier 1829) | Z71995 | AY169705 |  | Z70777 |  |  |
|  | Mullidae | *Mullus barbatus* Linnaeus 1758 |  |  |  | EF439552 | EF439143 |  |
|  |  | *Mullus surmuletus* Linnaeus 1758 | EF095566 | EF095594 |  | DQ197965 | EF095617 | EF095658 |
|  |  | *Upeneus moluccensis* (Bleeker 1855) |  |  |  | AF227675 |  | EU167747 |
|  | Nomeidae | *Psenes pellucidus* Lütken 1880 |  | AB205425 | AB205447 | AB205469 |  |  |
|  | Pinguipedidae | *Pinguipes brasilianus* Cuvier 1829 |  |  | EU074542 |  |  |  |
|  | Polyprionidae | *Polyprion americanus* (Bloch & Schneider 1801) | AM158291 | AY947616 | DQ107915 | EF392605 | EF427493 |  |
|  | Pomacentridae | *Abudefduf vaigiensis* (Quoy & Gaimard 1825) | AF436880 | AY365120 | AP006016 | AY208557 |  |  |
|  |  | *Chromis chromis* (Linnaeus 1758) |  | AF517577 |  | AY208527 |  | AY208640 |
|  | Pomatomidae | *Pomatomus saltatrix* (Linnaeus 1766) |  | AF055612 | DQ885112 | DQ080341 | DQ080430 | EU167741 |
|  | Priacanthidae | *Priacanthus hamrur* (Forsskål 1775) |  |  | DQ885115 |  |  | EU167865 |
|  | Rachycentridae | *Rachycentron canadum* (Linnaeus 1766) |  | DQ532949 | EF609446 | AB292793 |  | EU167910 |
|  | Scaridae | *Scarus ghobban* Forsskål 1775 |  |  | EF609452 |  |  |  |
|  |  | *Sparisoma cretense* (Linnaeus 1758) | SCU95777 | AF517578 |  | DQ198004 | DQ197906 | DQ457040 |
|  | Sciaenidae | *Argyrosomus regius* (Asso 1801) |  |  |  | DQ197924 | DQ197826 |  |
|  |  | *Umbrina canariensis* Valenciennes 1843 |  |  |  | EF392637 | EF427532 |  |
|  |  | *Umbrina cirrosa* (Linnaeus 1758) |  |  |  | AF143198 |  |  |
|  | Scombridae | *Acanthocybium solandri* (Cuvier 1832) | DQ854648 | DQ874727 | DQ835838 | DQ080324 | DQ874804 |  |
|  |  | *Auxis rochei* (Risso 1810) | AB176810 |  | DQ835852 | DQ080311 | DQ080400 |  |
|  |  | *Euthynnus alletteratus* (Rafinesque 1810) | AB176806 | DQ874730 | DQ835903 | DQ080308 | DQ080398 |  |
|  |  | *Katsuwonus pelamis* (Linnaeus 1758) | AB176808 | DQ874729 | DQ835922 | DQ080315 | DQ080410 |  |
|  |  | *Rastrelliger kanagurta* (Cuvier 1816) |  |  |  | DQ497857 |  |  |
|  |  | *Sarda sarda* (Bloch 1793) | DQ874691 | DQ874723 | DQ835917 | DQ080300 | DQ874800 |  |
|  |  | *Scomber japonicus* Houttuyn 1782 | AB241442 | EF458394 | EF433288 | AB018996 | AY141311 |  |
|  |  | *Scomber scombrus* Linnaeus 1758 | AB241438 | DQ874720 | DQ835839 | DQ080334 | DQ874797 | EU477493 |
|  |  | *Scomberomorus commerson* (Lacepède 1800) | EF095579 | EF095607 | DQ107670 | DQ497865 | EF095634 | EF095676 |
|  |  | *Scomberomorus tritor* (Cuvier 1832) | AF231582 | AF231539 |  | AF231666 |  |  |
|  |  | *Thunnus alalunga* (Bonnaterre 1788) | AB176804 |  | DQ835820 | DQ080289 | DQ080389 |  |
|  |  | *Thunnus thynnus* (Linnaeus 1758) | AY507951 |  | DQ835876 | DQ080266 | DQ080358 |  |
|  | Serranidae | *Epinephelus aeneus* (Geoffroy Saint-Hilaire 1817) | AY141367 | AY947593 |  | DQ197950 | AY141291 |  |
|  |  | *Epinephelus caninus* (Valenciennes 1843) | AM158294 | AY947585 |  | AJ420204 |  |  |
|  |  | *Epinephelus coioides* (Hamilton 1822) |  | AY947608 | DQ107891 | DQ354156 |  |  |
|  |  | *Epinephelus haifensis* (Ben-Tuvia 1853) |  |  |  | AJ420207 |  |  |
|  |  | *Epinephelus malabaricus* (Bloch & Schneider 1801) |  | DQ067309 | DQ107871 |  |  | AY551565 |
|  |  | *Epinephelus marginatus* (Lowe 1834) | AM158299 | AY947595 |  | AB179759 | DQ197854 |  |
|  |  | *Mycteroperca rubra* (Bloch 1793) | AM158292 | AY947587 |  | DQ197969 | DQ197871 |  |
|  |  | *Serranus atricauda* Günther 1874 | AM158286 |  |  | DQ197999 | EF439313 |  |
|  |  | *Serranus cabrilla* (Linnaeus 1758) | AM158283 |  |  | DQ198000 | EF439445 |  |
|  |  | *Serranus hepatus* (Linnaeus 1758) | AM158289 |  |  | EF439586 | EF439449 |  |
|  |  | *Serranus scriba* (Linnaeus 1758) | AM158288 |  |  | DQ198001 | EF439451 |  |
|  | Siganidae | *Siganus luridus* (Rüppell 1829) |  | DQ532959 |  | DQ898056 |  |  |
|  |  | *Siganus rivulatus* Forsskål & Niebuhr 1775 |  | DQ898115 |  | DQ898075 |  |  |
|  | Sillaginidae | *Sillago sihama* (Forsskål 1775) | EU257812 | EU257202 | EF607562 |  |  | EU167874 |
|  | Sparidae | *Boops boops* (Linnaeus 1758) |  | AF247396 |  | DQ197932 | EF439263 | EU167763 |
|  |  | *Crenidens crenidens* (Forsskål 1775) |  | AF247397 |  | AF240699 |  |  |
|  |  | *Dentex dentex* (Linnaeus 1758) |  | DQ532863 |  | AF143197 | EF427464 |  |
|  |  | *Dentex gibbosus* (Rafinesque 1810) |  | AJ247272 |  | DQ197941 | DQ197843 |  |
|  |  | *Dentex macrophthalmus* (Bloch 1791) |  | AJ247273 |  | EF392580 | EF427466 |  |
|  |  | *Dentex maroccanus* Valenciennes 1830 |  | EU410413 |  | DQ197942 | DQ197844 |  |
|  |  | *Diplodus annularis* (Linnaeus 1758) |  | AJ247286 |  | EF392581 | EF427467 |  |
|  |  | *Diplodus bellottii* (Steindachner 1882) |  | AJ247288 |  |  |  |  |
|  |  | *Diplodus cervinus* (Lowe 1838) |  | AF247420 |  | AF240723 | DQ197847 |  |
|  |  | *Diplodus puntazzo* (Walbaum 1792) |  | AJ247291 |  | EF392585 | EF427471 |  |
|  |  | *Diplodus sargus* (Linnaeus 1758) |  | AF365354 |  | EF427554 | DQ197848 |  |
|  |  | *Diplodus vulgaris* (Geoffroy Saint-Hilaire 1817) |  | AJ247294 |  | DQ197947 | DQ197849 |  |
|  |  | *Lithognathus mormyrus* (Linnaeus 1758) |  | AF247410 |  | AF240712 | DQ197863 | EU167782 |
|  |  | *Oblada melanura* (Linnaeus 1758) |  | AF247399 |  | AF240701 | EF439410 | EU167786 |
|  |  | *Pagellus acarne* (Risso 1827) |  | AF247411 |  | AF240713 | DQ197872 |  |
|  |  | *Pagellus bellottii* Steindachner 1882 |  | AF247412 |  | DQ197971 | DQ197873 |  |
|  |  | *Pagellus bogaraveo* (Brünnich 1768) | AY178432 |  |  | DQ197972 | DQ197874 |  |
|  |  | *Pagellus erythrinus* (Linnaeus 1758) |  | AJ247284 |  | DQ197973 | EF439417 | EU167790 |
|  |  | *Pagrus auriga* Valenciennes 1843 | AY178433 | AF247425 |  | DQ197974 | DQ197876 | EU167788 |
|  |  | *Pagrus caeruleostictus* (Valenciennes 1830) |  | AJ247276 |  | DQ197975 | DQ197877 | EU167789 |
|  |  | *Pagrus pagrus* (Linnaeus 1758) | AY178431 | AF247426 |  | DQ197976 | DQ197878 | EU167791 |
|  |  | *Sarpa salpa* (Linnaeus 1758) |  | AF247402 |  | DQ197992 | EF439306 | HQ676686 |
|  |  | *Sparus aurata* Linnaeus 1758 | EF095565 | AF247432 |  | AF240735 | EU224181 | EF095657 |
|  |  | *Spondyliosoma cantharus* (Linnaeus 1758) |  | AF247403 |  | AF240705 | EF439321 |  |
|  | Sphyraenidae | *Sphyraena sphyraena* (Linnaeus 1758) | AY141386 | DQ532964 |  | DQ080263 | AY141312 |  |
|  |  | *Sphyraena viridensis* Cuvier 1829 |  |  |  | DQ080262 | DQ080353 |  |
|  | Stromateidae | *Pampus argenteus* (Euphrasen 1788) | AY141383 | AY141453 | DQ107596 |  | AY141309 |  |
|  | Tetragonuridae | *Tetragonurus cuvieri* Risso 1810 |  | AB205429 | AB205451 | AB205473 |  |  |
|  | Trachinidae | *Echiichthys vipera* (Cuvier 1829) |  |  |  | EU492114 | EU492019 |  |
|  |  | *Trachinus draco* Linnaeus 1758 | AY141378 | AF518227 |  | EF439610 | AY141304 |  |
|  |  | *Trachinus radiatus* Cuvier 1829 |  |  |  | DQ198015 | EF439480 |  |
|  | Trachiuridae | *Lepidopus caudatus* (Euphrasen 1788) |  | AF100917 |  | DQ080261 | DQ080352 |  |
|  |  | *Trichiurus lepturus* Linnaeus 1758 | DQ874687 | AB201821 | EF607600 | DQ364151 | DQ874796 | EU167903 |
|  | Triterygiidae | *Tripterygion delaisi* Cadenat & Blache 1970 | AY098809 | AY098849 | AJ872120 |  |  |  |
|  |  | *Tripterygion melanurus* Guichenot 1850 |  | AJ868524 | AJ872145 |  |  |  |
|  |  | *Tripterygion tripteronotus* (Risso 1810) |  | AF324198 | AJ872130 |  |  |  |
|  | Uranoscopidae | *Uranoscopus scaber* Linnaeus 1758 | AF518213 |  |  | DQ198017 | EU036628 |  |
|  | Xiphiidae | *Xiphias gladius* Linnaeus 1758 | DQ854646 | DQ874734 | DQ107623 | DQ080249 | DQ874811 |  |
| Pleuronectiformes | Bothidae | *Arnoglossus imperialis* (Rafinesque 1810) | AF542209 | AY359651 |  |  | AY141283 |  |
|  |  | *Arnoglossus laterna* (Walbaum 1792) | AF542210 | AY359653 |  |  | EU224096 |  |
|  |  | *Arnoglossus thori* Kyle 1913 | AF542208 | AY157329 |  | AY029189 |  |  |
|  |  | *Bothus podas* (Delaroche 1809) | AF542221 | AY157326 |  | AF324334 | AY368313 |  |
|  | Citharidae | *Citharus linguatula* (Linnaeus 1758) | AF542220 | AY157325 |  | EF439510 | AY141323 |  |
|  | Pleuronectidae | *Platichthys flesus* (Linnaeus 1758) | AB125244 | AY359670 | EU524278 | AB125334 | EU492025 |  |
|  |  | *Pleuronectes platessa* Linnaeus 1758 | AF542207 | AY157328 |  | EU224075 | EU224175 |  |
|  | Scophthalmidae | *Lepidorhombus boscii* (Risso 1810) | AM931031 | DQ304652 |  | EF439534 | EF439124 |  |
|  |  | *Lepidorhombus whiffiagonis* (Walbaum 1792) | AY998042 | DQ195533 |  | EF427570 | EF439125 |  |
|  |  | *Psetta maxima* (Linnaeus 1758) | AF517557 | AY359664 |  | AY164471 | EU224174 |  |
|  |  | *Scophthalmus rhombus* (Linnaeus 1758) | AY998044 | AY359665 |  | EF427597 | EF439439 |  |
|  | Soleidae | *Bathysolea profundicola* (Vaillant 1888) |  | AY359659 |  |  |  |  |
|  |  | *Buglossidium luteum* (Risso 1810) |  | AY359663 |  | EU492126 | EU492030 |  |
|  |  | *Dicologlossa cuneata* (Moreau 1881) | AB125241 | AY157321 |  | AB125331 | EF456044 |  |
|  |  | *Microchirus azevia* (de Brito Capello 1867) | AB125238 | AY157318 |  | AB125329 | EF427488 |  |
|  |  | *Microchirus boscanion* (Chabanaud 1926) | AB125239 | AB125250 |  | AB125330 |  |  |
|  |  | *Microchirus hexophthalmus* (Bennett 1831) | AB125242 | AB125253 |  | AB125332 |  |  |
|  |  | *Microchirus ocellatus* (Linnaeus 1758) | AF542218 | AY157327 |  | AF113198 |  |  |
|  |  | *Microchirus variegatus* (Donovan 1808) | AF542215 | AY141429 |  | EF427582 | AY141284 |  |
|  |  | *Pegusa impar* (Bennett 1831) |  |  |  | AF113192 |  |  |
|  |  | *Pegusa lascaris* (Risso 1810) | AB125234 | AB125245 |  | AB125325 | EF427491 |  |
|  |  | *Solea aegyptiaca* Chabanaud 1927 |  |  |  | AF289718 |  |  |
|  |  | *Solea senegalensis* Kaup 1858 | AB125235 | AY359661 |  | AB125326 | EF439167 |  |
|  |  | *Solea solea* (Linnaeus 1758) | AF488492 | AF488442 |  | AB125327 | EU224131 | EF095644 |
|  |  | *Synaptura lusitanica* de Brito Capello 1868 | AB125243 | AB125254 |  | AB125333 | EF439470 |  |
|  |  | *Synapturichthys kleinii* (Risso 1827) | AB125237 | AB125248 |  | AB125328 | EF439468 |  |
| Scorpaeniformes | Cottidae | *Taurulus bubalis* (Euphrasen 1786) | AY141363 |  |  | EU492317 | EU492224 |  |
|  | Scorpaenidae | *Pontinus kuhlii* (Bowdich 1825) |  |  |  | DQ197983 | DQ197885 |  |
|  |  | *Pterois miles* (Bennett 1828) | DQ125237 | AJ429402 | EU148593 | EF209664 |  |  |
|  |  | *Scorpaena elongata* Cadenat 1943 |  |  |  | EF456020 | EF456081 |  |
|  |  | *Scorpaena maderensis* Valenciennes 1833 |  |  |  | DQ197996 | DQ197898 |  |
|  |  | *Scorpaena notata* Rafinesque 1810 | DQ125235 | AF518222 |  | DQ197997 | DQ197899 |  |
|  |  | *Scorpaena porcus* Linnaeus 1758 | DQ125238 |  |  | EF392615 | EU036590 |  |
|  |  | *Scorpaena scrofa* Linnaeus 1758 | DQ125234 | AF518223 |  | EU036494 | EF439442 |  |
|  |  | *Scorpaenodes arenai* Torchio 1962 | DQ125239 |  |  |  |  |  |
|  | Sebastidae | *Helicolenus dactylopterus* (Delaroche 1809) | DQ125236 | EU410418 | EF609371 | DQ197956 | DQ197858 |  |
|  |  | *Trachyscorpia cristulata* (Goode & Bean 1896) |  | AY538980 |  |  |  |  |
|  | Triglidae | *Chelidonichthys lucernus* (Linnaeus 1758) | AY141362 | EF120859 | EF609323 | EF427548 | AY141287 |  |
|  |  | *Eutrigla gurnardus* (Linnaeus 1758) |  |  |  | EF427560 | EF439111 |  |
|  |  | *Lepidotrigla cavillone* (Lacepède 1801) |  |  |  | EF439536 | EF439389 |  |
|  |  | *Trigla lyra* Linnaeus 1758 |  |  |  | EF439617 | EF439485 |  |
|  |  | *Trigloporus lastoviza* (Bonnaterre 1788) |  |  |  | EF427546 | EF439098 |  |
| Stomiiformes | Gonostomatidae | *Cyclothone braueri* Jespersen & Tåning 1926 | CY2MTSS04 |  |  |  |  |  |
|  |  | *Cyclothone pygmaea* Jespersen & Tåning 1926 | CY2MTSS15 | CY2MTLS31 |  |  |  |  |
|  |  | *Gonostoma denudatum* Rafinesque 1810 | AB026027 | AB026039 |  |  |  |  |
|  | Phosichthyidae | *Ichthyococcus ovatus* (Cocco 1838) |  |  | EU148211 |  |  | GQ860317 |
|  |  | *Vinciguerria poweriae* (Cocco 1838) |  |  |  |  |  | GQ860320 |
|  | Sternoptychidae | *Argyropelecus hemigymnus* Cocco 1829 |  | EU099497 | EU148087 |  |  |  |
|  |  | *Maurolicus muelleri* (Gmelin 1789) |  | AJ277245 | EU148246 |  |  |  |
|  |  | *Valenciennellus tripunctulatus* (Esmark 1871) |  |  |  |  |  | GQ860310 |
|  | Stomiidae | *Chauliodus sloani* Bloch & Schneider 1801 | AP002915 | AP002915 | EU148112 | AP002915 |  | GQ860327 |
|  |  | *Stomias boa boa* (Risso 1810) |  |  | EU148335 |  |  |  |
| Syngnathiformes | Centriscidae | *Macroramphosus scolopax* (Linnaeus 1758) | AY141354 | AY141424 | AP005988 | AP005988 | AY141280 |  |
|  | Fistulariidae | *Fistularia commersonii* Rüppell 1838 | AP005987 | AP005987 | EF607383 | AY786435 |  |  |
|  |  | *Fistularia petimba* Lacepède 1803 | AY141355 | AY141425 |  |  | AY141324 |  |
|  | Syngnathidae | *Entelurus aequoreus* (Linnaeus 1758) | AF354944 | DQ437522 | EU148160 | AF356044 |  |  |
|  |  | *Hippocampus fuscus* Rüppell 1838 |  | DQ288371 |  | DQ288354 |  |  |
|  |  | *Hippocampus hippocampus* (Linnaeus 1758) |  | DQ288358 |  | AF192665 |  |  |
|  |  | *Hippocampus ramulosus* Leach 1814 | AY368288 | AY368310 |  |  | AY368330 |  |
|  |  | *Nerophis ophidion* (Linnaeus 1758) | AF354943 | AF354994 |  | AF356043 |  |  |
|  |  | *Syngnathus abaster* Risso 1827 | AF354959 | AF355010 |  | AF356060 |  |  |
|  |  | *Syngnathus acus* Linnaeus 1758 | AF354940 | AF354991 |  | AF356040 |  |  |
|  |  | *Syngnathus rostellatus* Nilsson 1855 | AF354941 | AF354992 |  | AF356041 |  |  |
|  |  | *Syngnathus taenionotus* Canestrini 1871 | AF354960 | AF355011 |  | AF356061 |  |  |
|  |  | *Syngnathus typhle* Linnaeus 1758 | AY368291 | AF354993 |  | AF356042 | AY368326 |  |
| Tetraodontiformes | Caproidae | *Capros aper* (Linnaeus 1758) | EF095553 | DQ532846 | EU148107 | AP009159 | AY141262 | EF095638 |
|  | Molidae | *Mola mola* (Linnaeus 1758) | AY700258 | DQ532911 | AP006238 | AY940835 | AF137215 | EF095643 |
|  |  | *Ranzania laevis* (Pennant 1776) | AP006047 | AP006047 | DQ521011 | EF392608 | EF427496 |  |
|  | Tetraodontidae | *Lagocephalus sceleratus* (Gmelin 1789) |  | AB194240 |  | EF362414 |  |  |
|  |  | *Lagocephalus spadiceus* (Richardson 1845) |  |  | EF60741 9 |  |  |  |
|  |  | *Sphoeroides pachygaster* (Müller & Troschel 1848) | AP006745 | AB194239 | EU074598 | EF392642 | EF427517 |  |
|  |  | *Sphoeroides spengleri* (Bloch 1785) | AY700284 | AY679668 |  |  |  | AY700354 |
|  |  | *Takifugu rubripes* (Temminck & Schlegel 1850) | NC_004299.1 | NC_004299.1 | NC_004299.1 | NC_004299.1 | AF137214.1 | AY700363 |
|  |  | *Tetraodon nigroviridis* Marion de Procé 1822 | NC_007176.1 | NC_007176.1 | NC_007176.1 | NC_007176.1 | AJ293018.1 |  |
| Zeiformes | Zeidae | *Zeus faber* Linnaeus 1758 | AF149993 | DQ027916 | EF609496 | DQ198019 | EF439493 | FJ215202 |

For each species represented in the phylogeny we have listed the GenBank accession number of each gene used in the phylogenetic analysis. An empty cell represents a gene that was not included in the analysis. Species names, corresponding name authorities and classification follow FishBase version 02/2011 (<http://www.fishbase.org/>).
