# Supplementary material for: A Phylogenetic Perspective on the Evolution of Mediterranean Teleost Fishes
Source: PLoS One. 2012 May 8;7(5):e36443. doi: 10.1371/journal.pone.0036443 (PMC3348158; doi:10.1371/journal.pone.0036443)
Supplement: Appendix S2 — Gene representation and saturation in the phylogenetic analysis. (DOC) [file pone.0036443.s002.doc]

**Appendix 2 Summary of gene representation and saturation in the phylogenetic analysis.**

In this appendix we provide a summary of representation for each gene, as well as an analysis of saturation by gene.

*Gene representation*

Even though the percent of species represented solely by mitochondrial genes is large, more than half of the species in the phylogeny are represented by some combination of nuclear and mitochondrial genes (Table A2.1). The least represented gene is RAG1 with 80 species, followed by COXI, with 118 species (Figure A2.1). The best represented gene is 16S, with 265 species (Figure A2.1). The phylogeny contains a total of 373 species, so these numbers correspond to a minimum of 21 % and a maximum of 71 % respectively. Moreover, whereas 16% of the species are represented by only 1 gene, and 5 % are represented by all 6 genes, the vast majority are represented by at least 2 genes (84 %) (Figure A2.2).

**Table A2.1** **Number of cases and corresponding percent (based on the total number of species in the phylogeny) where the species was represented by nuclear versus mitochondrial genes.**

|  | Number of species | Percent |
| --- | --- | --- |
| Only mitochondrial genes | 154 | 41.3 |
| Only nuclear genes | 13 | 3.5 |
| Some combination of nuclear and mitochondrial genes | 206 | 55.2 |
| Only 1 nuclear gene | 175 | 46.9 |
| Both RAG1 and RHOD genes | 44 | 11.8 |

**Figure A2.1**

Number of species represented for each gene (based only on the 373 species represented in the phylogeny).

**
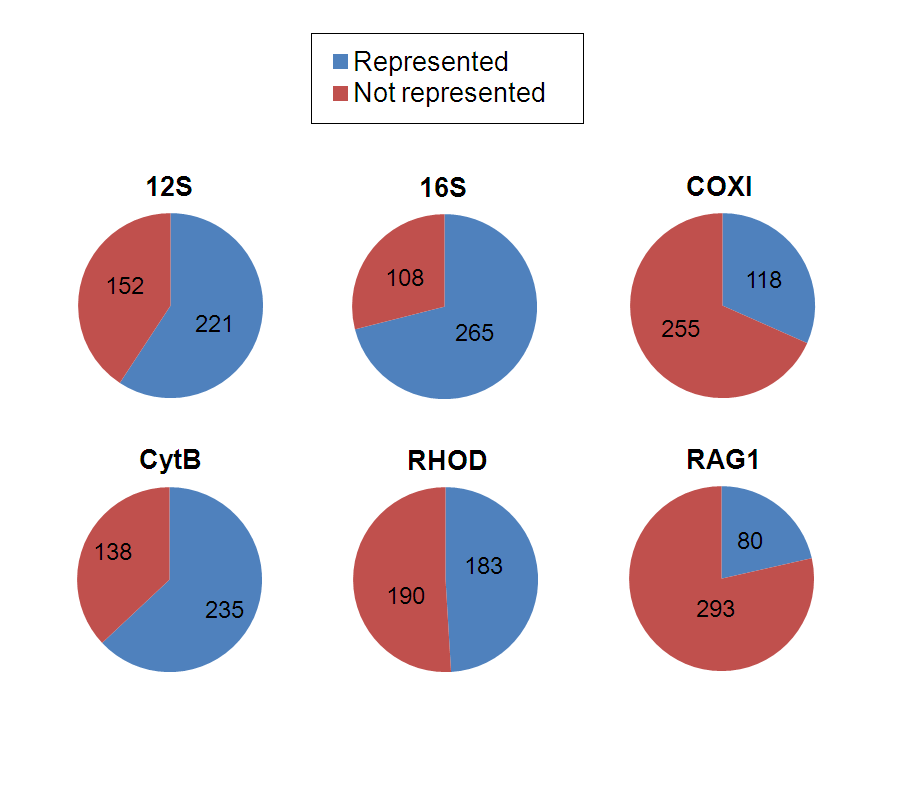
**

**Figure A2.2**

Number of species represented by 1, 2, 3, 4, 5 or 6 genes (based on the 373 species represented in the phylogeny), irrespective of whether they are nuclear or mitochondrial.


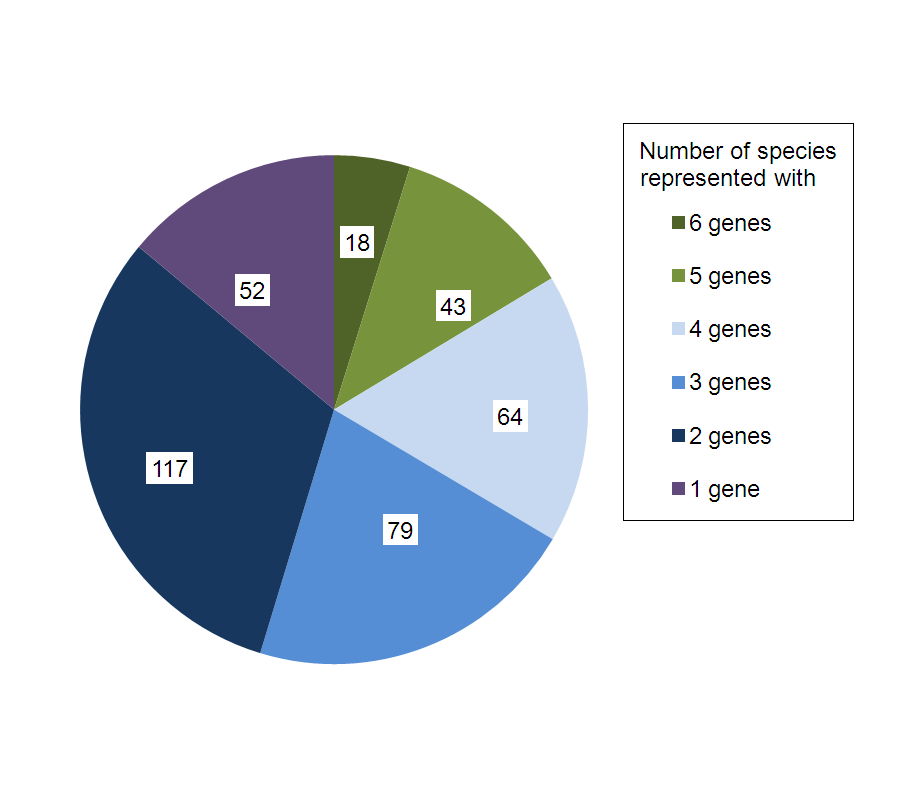


*Saturation information by gene*

Here we compared saturation of the nucleotide substitutions in the two nuclear recombination activating gene 1 (RAG1) and rhodopsin (RHO) markers and 4 mitochondrial cytochrome b (CYB), 12S rRNA, 16rRNA, and cytochrome c oxidase subunit 1 (COX1) markers when inferring the phylogeny of Mediterranean teleosts.

To evaluate whether the slower-evolving RAG1 and RHO and the faster-evolving CYB, 12S rRNA, 16S rRNA, and COX1 saturated when reconstructing the teleost phylogeny, we constructed saturation-plots of the number of maximum likelihood inferred substitutions between any pair of taxa (i.e., patristic distances measured on the highest-likelihood phylogram reconstructed from each of the 6 alignments) against the corresponding observed (apparent) number of nucleotide differences in the 6 alignments. The slope of the regression lines for example suggest that the saturation level of the RAG1 marker is moderate, whereas the COX1 display stronger saturation. The former will provide phylogenetic information for deeper nodes in the Mediterranean teleost tree, whereas the latter will provide information for terminal nodes.

| 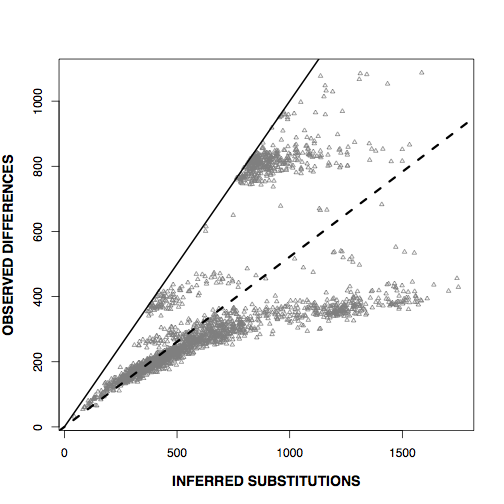 | 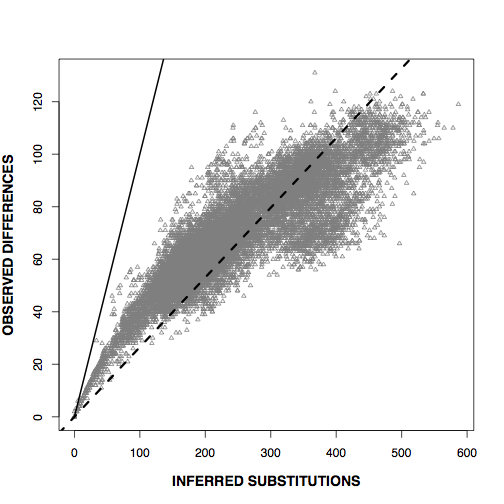 |
| --- | --- |
| **Saturation plot of the RAG1 marker**.  Dashes correspond to the regression line through the origin (slope = 0.52). | **Saturation plot of the RHO marker**.  Dashes correspond to the regression line through the origin (slope = 0.26). |
| **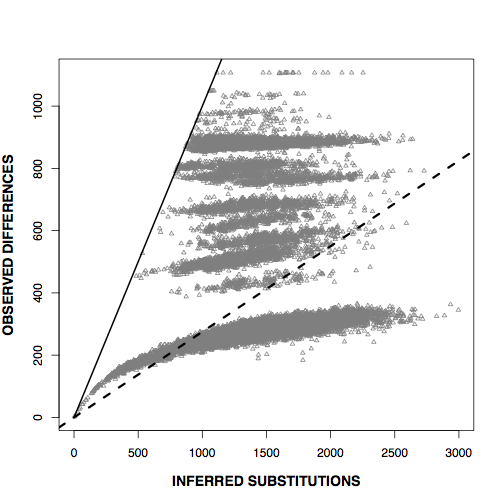** | **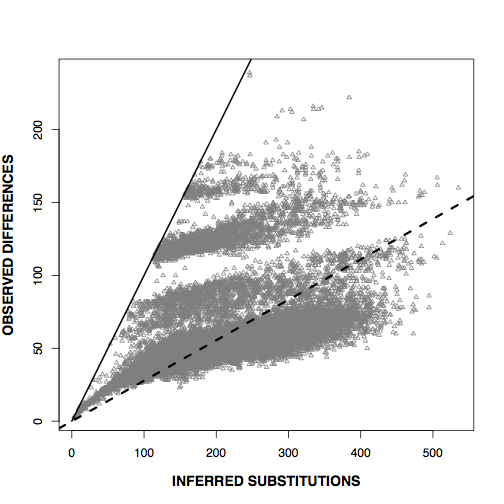** |
| **Saturation plot of the CYB marker**.  Dashes correspond to the regression line through the origin (slope = 0.28). | **Saturation plot of the 12S rRNA marker**.  Dashes correspond to the regression line through the origin (slope = 0.28). |
| **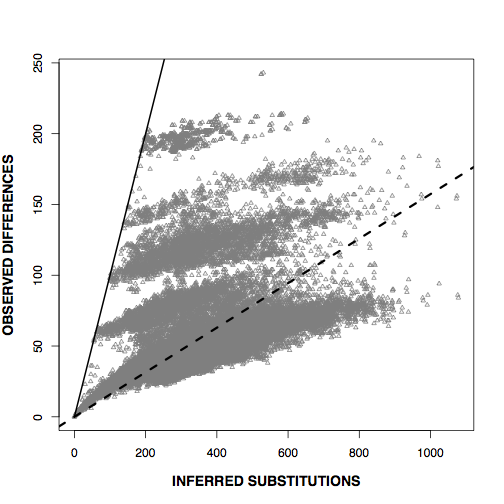** | 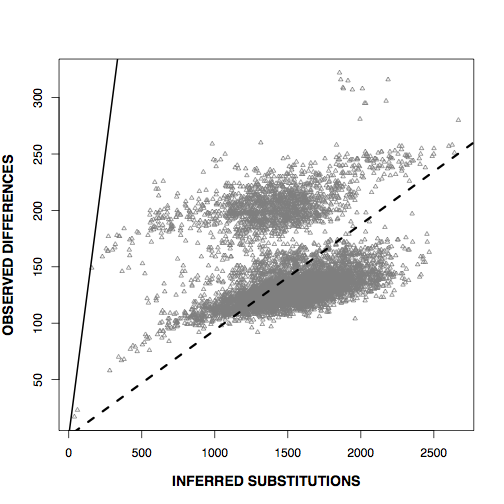 |
| **Saturation plot of the 16SrRNA marker**.  Dashes correspond to the regression line through the origin (slope = 0.16). | **Saturation plot of the COX1 marker**.  Dashes correspond to the regression line through the origin (slope = 0.09). |
| The straight line indicates the absence of saturation, i.e., the situation for which the number of inferred substitutions is equal to the number of observed differences in the alignment. Note the difference of X-axis scale between the six plots. | |
