# Supplementary material for: A Phylogenetic Perspective on the Evolution of Mediterranean Teleost Fishes
Source: PLoS One. 2012 May 8;7(5):e36443. doi: 10.1371/journal.pone.0036443 (PMC3348158; doi:10.1371/journal.pone.0036443)
Supplement: Appendix S3 — Species grafted at their most recent common ancestor (MRCA). (DOC) [file pone.0036443.s003.doc]

**Appendix S3:** **Species attached to their most recent common ancestor (MRCA).** Table S3.1 shows a summary of the level at which the species was attached to the raw chronogram, whereas Table S3.2 shows the list of species attached. Name authorities were taken from FishBase v02/2011.

**Table S3.1 Summary of number of species attached to the raw chronogram.**

|  | At least two congeners (C2) | At least one congener (C1) | Family | Order | Total |
| --- | --- | --- | --- | --- | --- |
| All Species | 33 | 10 | 57 | 24 | 124 |
| Endemics | 9 | 2 | 16 | 4 | 31 |
| Non-endemic natives | 16 | 6 | 28 | 12 | 62 |
| Exotics | 8 | 2 | 13 | 8 | 31 |

**Table S3.2** List of species attached to the raw chronogram.

| **Order** | **Family** | **Species Name** | **Status** | **Attachment Level** |
| --- | --- | --- | --- | --- |
| Anguilliformes | Chlopsidae | *Chlopsis bicolor* Rafinesque 1810 | Native | O |
|  | Congridae | *Ariosoma balearicum* (Delaroche 1809) | Native | O |
|  |  | *Conger conger* (Linnaeus 1758) | Native | O |
|  |  | *Gnathophis mystax* (Delaroche 1809) | Native | O |
|  | Heterenchelyidae | *Panturichthys fowleri* (Ben-Tuvia 1953) | Endemic | O |
|  | Ophichthidae | *Apterichtus anguiformis* (Peters 1877) | Native | F |
|  |  | *Apterichtus caecus* (Linnaeus 1758) | Native | F |
|  |  | *Dalophis imberbis* (Delaroche 1809) | Native | F |
|  |  | *Ophichthus rufus* (Rafinesque 1810) | Endemic | F |
|  |  | *Ophisurus serpens* (Linnaeus 1758) | Native | F |
|  |  | *Pisodonophis semicinctus* (Richardson 1848) | Exotic | F |
|  | Synaphobranchidae | *Dysomma brevirostre* (Facciolà 1887) | Native | O |
| Aulopiformes | Evermannellidae | *Evermannella balbo* (Risso 1820) | Native | O |
|  | Paralepididae | *Paralepis speciosa* Belloti 1878 | Endemic | F |
| Beloniformes | Exocoetidae | *Cheilopogon furcatus* (Mitchill 1815) | Exotic | F |
|  |  | *Cheilopogon heterurus* (Rafinesque 1810) | Native | F |
|  |  | *Exocoetus obtusirostris* Günther 1866 | Native | F |
|  |  | *Parexocoetus mento* (Valenciennes 1847) | Exotic | F |
|  | Hemiramphidae | *Hyporhamphus picarti* (Valenciennes 1847) | Native | C1 |
| Clupeiformes | Clupeidae | *Herklotsichthys punctatus* (Rüppell 1837) | Exotic | F |
| Gadiformes | Macrouridae | *Nezumia sclerorhynchus* (Valenciennes 1838) | Native | F |
|  | Moridae | *Eretmophorus kleinenbergi* Giglioli 1889 | Native | F |
|  |  | *Gadella maraldi* (Risso 1810) | Native | F |
|  |  | *Lepidion guentheri* (Giglioli 1880) | Exotic | F |
|  |  | *Lepidion lepidion*  (Risso 1810) | Endemic | F |
|  |  | *Physiculus dalwigki* Kaup 1858 | Native | F |
| Lophiiformes | Chaunacidae | *Chaunax pictus* Lowe 1846 | Exotic | O |
| Mugiliformes | Mugilidae | *Liza carinata* (Valenciennes 1836) | Exotic | C2 |
| Myctophiformes | Myctophidae | *Diaphus holti* Tåning 1918 | Native | C2 |
|  |  | *Lampanyctus crocodilus* (Risso 1810) | Native | F |
|  |  | *Lampanyctus pusillus* (Johnson 1890) | Native | F |
|  |  | *Notoscopelus elongatus* (Costa 1844) | Endemic | C1 |
| Osmeriformes | Alepocephalidae | *Alepocephalus rostratus* Risso 1820 | Native | O |
|  | Argentinidae | *Glossanodon leioglossus* (Valenciennes 1848) | Native | F |
|  | Microstomatidae | *Nansenia iberica* Matallanas 1985 | Endemic | O |
|  |  | *Nansenia oblita* (Facciolà, 1887) | Native | O |
|  |  | *Microstoma microstoma* (Risso 1810) | Native | O |
| Perciformes | Apogonidae | *Apogon pharaonis* (Belloti 1874) | Exotic | C1 |
|  | Blenniidae | *Hypleurochilus bananensis* (Poll 1959) | Native | F |
|  |  | *Salaria basilisca* (Valenciennes 1836) | Endemic | C1 |
|  | Callionymidae | *Callionymus fasciatus* Valenciennes 1837 | Native | C2 |
|  |  | *Callionymus filamentosus* Valenciennes 1837 | Exotic | C2 |
|  |  | *Callionymus pusillus* Delaroche 1809 | Native | C2 |
|  |  | *Callionymus risso* Lesueur 1814 | Native | C2 |
|  |  | *Synchiropus phaeton* (Günther 1861) | Native | F |
|  | Carangidae | ***Campogramma glaycos*** (**Lacepède 1801)** | Native | F |
|  |  | ***Naucrates ductor*** (**Linnaeus 1758)** | Native | F |
|  | Centracanthidae | *Centracanthus cirrus* Rafinesque 1810 | Native | F |
|  | Centrolophidae | *Schedophilus medusophagus* (Cocco 1839) | Native | F |
|  | Echeneidae | *Remora brachyptera* (Lowe 1839) | Native | C2 |
|  | Epigonidae | *Epigonus denticulatus* Dieuzeide 1950 | Native | C2 |
|  |  | *Microichthys coccoi* Rüppell 1852 | Endemic | F |
|  |  | *Microichthys sanzoi* Sparta 1950 | Endemic | F |
|  | Gobiidae | *Buenia jeffreysii* (Günther 1867) | Native | C1 |
|  |  | *Chromogobius quadrivittatus* (Steindachner 1863) | Endemic | F |
|  |  | *Chromogobius zebratus* (Kolombatovic 1891) | Endemic | F |
|  |  | *Corcyrogobius liechtensteini* (Kolombatovic 1891) | Endemic | F |
|  |  | *Deltentosteus collonianus* (Risso 1820) | Native | F |
|  |  | *Deltentosteus quadrimaculatus* (Valenciennes 1837) | Native | F |
|  |  | *Didogobius bentuvii* Miller 1966 | Endemic | F |
|  |  | *Didogobius schlieweni* Miller 1993 | Endemic | F |
|  |  | *Didogobius splechtnai* Ahnelt & Patzner 1995 | Endemic | F |
|  |  | *Gammogobius steinitzi* Bath 1971 | Endemic | F |
|  |  | *Gobius ater* Bellotti 1888 | Endemic | C2 |
|  |  | *Gobius couchi* Miller & El-Tawil 1974 | Exotic | C2 |
|  |  | *Gobius fallax* Sarato 1889 | Endemic | C2 |
|  |  | *Gobius geniporus* Valenciennes 1837 | Endemic | C2 |
|  |  | *Gobius roulei* de Buen 1928 | Native | C2 |
|  |  | *Gobius strictus* Fage 1907 | Endemic | C2 |
|  |  | *Gobius vittatus* Vinciguerra 1883 | Endemic | C2 |
|  |  | *Lebetus guilleti* (Le Danois 1913) | Native | F |
|  |  | *Millerigobius macrocephalus* (Kolombatovic 1891) | Endemic | F |
|  |  | *Monishia ochetica* (Norman 1927) | Exotic | F |
|  |  | *Odondebuenia balearica* (Pellegrin & Fage 1907) | Endemic | F |
|  |  | *Oxyurichthys papuensis* (Valenciennes 1837) | Exotic | F |
|  |  | *Pomatoschistus bathi* Miller 1982 | Endemic | C2 |
|  |  | *Pomatoschistus tortonesei* Miller 1969 | Endemic | C2 |
|  |  | *Silhouettea aegyptia* (Chabanaud 1933) | Exotic | F |
|  |  | *Speleogobius trigloides* Zander & Jelinek 1976 | Endemic | F |
|  |  | *Thorogobius ephippiatus* (Lowe 1839) | Native | F |
|  |  | *Thorogobius macrolepis* (Kolombatovic 1891) | Endemic | F |
|  |  | *Vanneaugobius pruvoti* (Fage 1907) | Native | F |
|  | Labridae | *Pteragogus pelycus* Randall 1981 | Exotic | F |
|  | Mullidae | *Pseudupeneus prayensis* (Cuvier 1829) | Exotic | F |
|  |  | *Upeneus asymmetricus* Lachner 1954 | Exotic | C1 |
|  | Nomeidae | *Cubiceps capensis* (Smith 1845) | Native | F |
|  | Sciaenidae | *Sciaena umbra* Linnaeus 1758 | Native | F |
|  |  | *Umbrina ronchus* Valenciennes 1843 | Native | C2 |
|  | Scombridae | *Orcynopsis unicolor* (Geoffroy Saint-Hilaire 1817) | Native | F |
|  | Serranidae | *Anthias anthias* (Linnaeus 1758) | Native | F |
|  |  | *Epinephelus alexandrinus* (Forsskål 1775) | Native | C2 |
|  | Sparidae | *Rhabdosargus haffara* (Forsskål 1775) | Exotic | F |
|  | Sphyraenidae | *Sphyraena chrysotaenia* Klunzinger 1884 | Exotic | C2 |
|  |  | *Sphyraena flavicauda* Rüppell 1838 | Exotic | C2 |
|  | Trachinidae | *Trachinus araneus* Cuvier 1829 | Native | C2 |
| Pleuronectiformes | Bothidae | *Arnoglossus kessleri* Schmidt 1915 | Endemic | C2 |
|  |  | *Arnoglossus rueppelii* (Cocco 1844) | Native | C2 |
|  | Cynoglossidae | *Cynoglossus sinusarabici* (Chabanaud 1931) | Exotic | O |
|  |  | *Symphurus ligulatus* (Cocco 1844) | Native | O |
|  |  | *Symphurus nigrescens* Rafinesque 1810 | Native | O |
|  | Pleuronectidae | *Platichthys flesus* (Linnaeus 1758) | Native | C2 |
|  | Soleidae | *Pegusa nasuta* (Pallas 1814) | Native | C2 |
| Scorpaeniformes | Liparidae | *Eutelichthys leptochirus* **Tortonese 1959** | Endemic | O |
|  |  | *Paraliparis murieli* Matallanas 1984 | Endemic | O |
|  | Peristediidae | *Peristedion cataphractum* (Linnaeus 1758) | Native | O |
|  | Platycephalidae | *Papilloculiceps longiceps* (Cuvier 1829) | Exotic | O |
|  |  | *Platycephalus indicus* (Linnaeus 1758) | Exotic | O |
|  |  | *Sorsogona prionota* (Sauvage 1873) | Exotic | O |
|  | Scorpaenidae | *Scorpaena loppei* Cadenat 1943 | Native | C2 |
|  |  | *Scorpaena stephanica* Cadenat 1943 | Exotic | C2 |
|  | Triglidae | *Lepidotrigla dieuzeidei* Blanc & Hureau 1973 | Native | C1 |
| Stomiiformes | Phosichthyidae | *Vinciguerria attenuata* (Cocco 1838) | Native | C1 |
|  | Sternoptychidae | *Valenciennellus tripunctulatus* (Esmark 1871) | Native | F |
|  | Stomiidae | *Bathophilus nigerrimus* Giglioli 1882 | Native | F |
| Syngnathiformes | Syngnathidae | *Nerophis maculatus* **Rafinesque, 1810** | Native | C1 |
|  |  | *Minyichthys sentus* **Dawson, 1982** | Native | F |
|  |  | *Syngnathus phlegon* Risso 1827 | Native | C2 |
|  |  | *Syngnathus tenuirostris* Rathke 1837 | Endemic | C2 |
| Tetraodontiformes | Diodontidae | *Diodon hystrix* Linnaeus 1758 | Exotic | O |
|  | Monacanthidae | *Stephanolepis diaspros* **Fraser-Brunner 1940** | Exotic | O |
|  | Ostraciidae | *Tetrosomus gibbosus* (Linnaeus 1758) | Exotic | O |
|  | Tetraodontidae | *Lagocephalus lagocephalus* (Linnaeus 1758) | Exotic | C2 |
|  |  | *Lagocephalus suezensis* Clark & Gohar 1953 | Exotic | C2 |
|  |  | *Torquigener flavimaculosus* Hardy & Randall 1983 | Exotic | F |
|  |  | *Tylerius spinosissimus* (Regan 1908) | Exotic | F |

Species grafted into the final chronogram next to their nearest closest relative for the diversification analyses. Status: species were classified as endemic, (non-endemic) native or exotic. Attachment level: species were attached to a congener if there were at least two congeners present in the phylogeny (C2); if there was only one congener present in the phylogeny (C2), they were attached to the nearest node joining the congener and the closest species in the phylogeny; if no congener was present, the new species was attached to the most recent common ancestor of the same family (F) or of the same order (O), i.e. to the node joining all members of the same family or order. Each one of these attachments levels was carried out sequencially one after the other, in four different and increasingly more species rich chronograms. Species names, the corresponding name authorities and classification follow FishBase version 02/2011 (<http://www.fishbase.org/>).
